# Co_3_O_4_ Quantum Dots Intercalation Liquid‐Crystal Ordered‐Layered‐Structure Optimizing the Performance of 3D‐Printing Micro‐Supercapacitors

**DOI:** 10.1002/advs.202303636

**Published:** 2023-09-26

**Authors:** Huijie Zhou, Yangyang Sun, Hui Yang, Yijian Tang, Yiyao Lu, Zhen Zhou, Shuai Cao, Songtao Zhang, Songqing Chen, Yizhou Zhang, Huan Pang

**Affiliations:** ^1^ School of Chemistry and Chemical Engineering Yangzhou University Yangzhou Jiangsu 225009 P. R. China; ^2^ Institute of Advanced Materials and Flexible Electronics (IAMFE) School of Chemistry and Materials Science Nanjing University of Information Science and Technology Nanjing 210044 P. R. China

**Keywords:** 3D printing, Co_3_O_4_‐quantum dots, liquid‐crystal nanocomposite, micro‐supercapacitors, ordered‐layered‐structure

## Abstract

The effects of near surface or surface mechanisms on electrochemical performance (lower specific capacitance density) hinders the development of 3D printed micro supercapacitors (MSCs). The reasonable internal structural characteristics of printed electrodes and the appropriate intercalation material can effectively compensate for the effects of surface or near‐surface mechanisms. In this study, a layered structure is constructed inside an electrode using an ink with liquid‐crystal characteristics, and the pore structure and oxidation active sites of the layered electrode are optimized by controlling the amount of Co_3_O_4_‐quantum dots (Co_3_O_4_ QDs). The Co_3_O_4_ QDs are distributed in the pores of the electrode surface, and the insertion of Co_3_O_4_ QDs can effectively compensate for the limitations of surface or near‐surface mechanisms, thus effectively improving the pseudocapacitive characteristics of the 3D‐printed MSCs. The 3D printed MSC exhibits a high area capacitance (306.13 mF cm^−2^) and energy density (34.44 µWh cm^−2^ at a power density of 0.108 mW cm^−2^). Therefore, selecting the appropriate materials to construct printable electrode structures and effectively adjusting material ratios for efficient 3D printing are expected to provide feasible solutions for the construction of various high‐energy storage systems such as MSCs.

## Graphical abstract

Co_3_O_4_ QDs are distributed in the pores on the electrode surface, and the internal structural porosity of the electrode is adjusted by adjusting the insertion amount of QDs. The insertion of Co_3_O_4_ QDs can effectively compensate for the limitations of surface or near surface mechanisms, thereby effectively improving the pseudo‐capacitance characteristics of 3D‐printed MSCs.

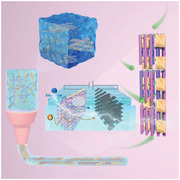

## 1 Introduction

The continuous increase in energy demand for intelligent, portable, and miniaturized electronic products has promoted the development of micro energy storage devices.^[^ ^1^, ^2^, ^3^, ^4^, ^5^, ^6^ ^]^ Microscale planar electrochemical energy storage devices with excellent electrochemical performances have become the ideal energy storage device.^[^ ^7^, ^8^, ^9^, ^10^, ^11^ ^]^ As a typical electrochemical energy storage device with high power density, excellent charge‐discharge reversibility, and reliability, supercapacitors (SCs) have demonstrated significant potential in energy storage.^[^ ^12^, ^13^ ^]^ However, their low energy densities severely hinder their widespread development and application in energy storage devices. The construction methods of SCs (including immersion coating, laser engraving, blade unfolding, and mask‐assisted filtering) have advanced substantially; however, inherent drawbacks remain, such as cumbersome manufacturing steps, low efficiency, and limited material utilization, which hinder the precise and effective large‐scale development of their manufacture.^[^ ^14^, ^15^, ^16^, ^17^, ^18^, ^19^ ^]^ Micro‐SCs (MSCs) are promising planar electrochemical energy storage devices that have achieved widespread attention owing to their excellent electrochemical performance.^[^ ^20^, ^21^, ^22^ ^]^ However, their low operating voltage and capacity caused by surface or near‐surface mechanisms severely hinder their rapid development in electrochemistry. Research has shown that the growth of metal oxides on the surface of printed electrodes through the 3D printing of carbon‐based material skeletons and electrodeposition can effectively improve the pseudocapacitance characteristics of the materials. However, this process is complex and limited by solvents. Therefore, suitable 3D printing materials for the insertion and construction of suitable electrode structures must be identified.^[^ ^23^, ^24^, ^25^ ^]^ MSCs generally appear in two configurations: symmetric and asymmetric. Symmetric MSCs have a limited device voltage because of water splitting.^[^ ^26^ ^]^ To avoid these limitations and improve the energy storage performance, constructing asymmetric MSCs with 3D structures is a promising approach.^[^ ^27^ ^]^ In addition, for MSCs, the weight of the electrodes is not an appropriate metric for evaluating their performance. Therefore, within a limited footprint, the rational design of electrode structures and the selection of materials are critical for improving the area and volume performances.

Most MSC manufacturing processes are based on photolithography technology and are limited by the necessity for high‐cost manufacturing techniques to achieve current collector or microelectrode modes.^[^ ^28^, ^29^, ^30^ ^]^ 3D printing is a promising method owing to its advantages of controllable structural manipulation and rapid prototyping.^[^ ^31^, ^32^, ^33^ ^]^ A reasonable selection of electrode materials is crucial for regulating the electrochemical performance of 3D printed MSCs.^[^ ^34^ ^]^ Typical printable electrode materials include 0D structured metal oxide nanoparticles, 1D structured silver‐nanowires (NWs), metal oxide NWs, carbon nanotubes (CNTs), 2D structured metal oxide nanosheets, graphene oxide (GO), and MXene. Two main electrode materials have been widely developed and utilized: carbon materials (such as 1D CNTs and 2D graphene nanosheets) and pseudocapacitive materials with Faraday charge storage (such as transition metal oxides). The former materials are widely used in micro‐electrical double‐layer capacitors. The latter materials are typically used to achieve higher capacitances.^[^ ^35^, ^36^, ^37^ ^]^ Although carbon materials are the most widely used elements, transition metal oxides can provide higher energy densities. Co_3_O_4_ has received widespread attention as a promising active material for SCs owing to its high theoretical capacitance, outstanding stability, excellent reversible redox performance, low cost, and environmental friendliness. However, Co_3_O_4_ has a lower conductivity and is less stable compared with carbon materials.^[^ ^38^ ^]^ The ability of multivalent vanadium oxides to undergo rapid redox reactions promotes rapid charge transfer. 0D quantum dots (QDs) have been widely used in many fields owing to their advantages such as large specific surface area, small volume, and high surface activity. As a 3D printing ink material, 0D metal oxide QDs present the characteristics of easy dispersion, printability, and pore structure adjustment. Therefore, conductive carbon and pseudocapacitive metal oxides with different dimensions have been integrated into the above‐mentioned printable ink to create a hybrid electrode, which increases the energy density while maintaining a high power density.^[^ ^39^, ^40^ ^]^ In addition, conductivity can be improved by constructing an optimized electrode structure with fast kinetic channels and oxidation active sites on the electrode. This significantly improves the overall electrochemical performance of the MSCs.

**Figure 1:**
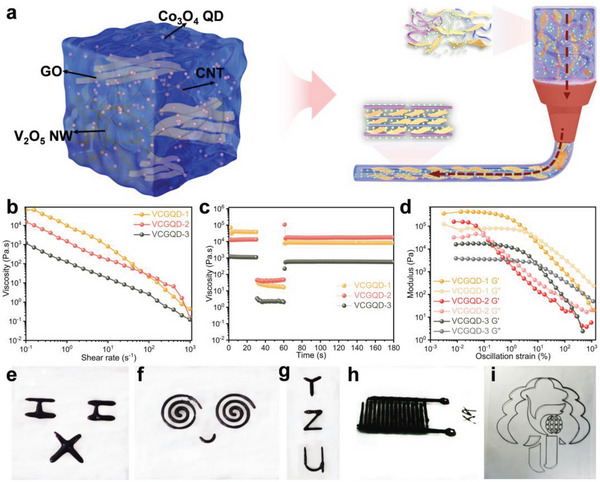
a) Schematic diagram of manufacturing process of VCGQDs hydrogel electrode structure to be 3D printed; b) The functional relationship between the apparent viscosity of the prepared VCGQD ink and shear rate; c) PHS experiment; d) The relationship between the storage modulus (G′) and loss modulus (G″) of VCGQD ink and oscillatory strain; e–i) Print digital photos of VCGQD ink of different shapes.

Herein, we introduce an electrode structure that utilizes 3D printed shear fields to construct an ordered porous layered structure and adjust the pseudo‐capacitive electrochemical properties of MSCs by inserting Co_3_O_4_ QDs into the layered structure. A high‐resolution interdigital electrode based on extrusion 3D printing is prepared using GO, CNTs, and V_2_O_5_ NWs and Co_3_O_4_ QDs pseudo plastic nanocomposite ink (**Figure** **1**a). The inner layer of the electrode is constructed using ink with liquid‐crystal characteristics. The pore structure and active oxidation sites of the layered electrode are adjusted based on the amount of Co_3_O_4_ QDs. The MSC cathode material is constructed using V_2_O_5_ NWs and Co_3_O_4_ QDs as active materials and CNTs and GO as conductive fillers; meanwhile, MXene is used to construct a counter electrode for an asymmetric MSC. The MSC of this structure offers an area specific capacitance of 306.13 mF cm^−2^ at a current density of 0.24 mA cm^−2^, and an energy density of 34.44 µWh cm^−2^ at a power density of 0.108 mW cm^−2^. To further demonstrate that the insertion of Co_3_O_4_ QDs can effectively improve the electrochemical performance of the material, we incorporate carbon dots (CDs) into it and characterize them in a series. The optimization of this structural configuration promotes the design of printed electrode materials and structures. This study provides new ideas for the development and application of high‐performance microelectrode electrochemical energy storage device.

## 2 Results and Discussion

In 3D printing technology, the extrudability, filament formation, shape accuracy, and degree of ink preservation depend on the viscosity and rheological properties of the ink. First, the rheological properties of different inks were investigated to examine their applicability in 3D printing. At a shear rate of 0.1 s^−1^, the viscosity of the mixed gel reached its maximum value and the viscosity gradually decreased as the shear rate increased (0.1–1000 s^−1^), thus demonstrating the shear‐thinning behavior of non‐Newtonian fluids (Figure 1b). This shear‐thinning behavior is conducive to the continuous and smooth extrusion of ink from microsized needles. To elucidate the shear‐dilution behavior of the gel during 3D printing, peak‐hold‐step experiments were performed to simulate an extrusion‐based printing process (Figure 1c). When the shear rate increased rapidly from 0.1 to 100 s^−1^ and the extrusion step was simulated for 30 s at a shear rate of 100 s^−1^, the viscosity of gel decreased immediately. However, when the shear rate returned to a low shear rate of 0.1 s^−1^, the viscosity returned swiftly to a high level. Nanocomposite gels exhibiting both high elasticity and high viscosity enable 3D printing based on extrusion, thus allowing high‐resolution and complex patterns to be generated rapidly. The viscoelastic fingerprint of the mixed gel shows that the storage modulus (G′) exceeds the loss modulus (G″) in the platform area. At this time, the mixed gel mainly showed solid behavior, thus indicating the formation of a network composed of highly permeable GO, CNTs, V_2_O_5_ NWs, and Co_3_O_4_ QDs (VCGQDs) (Figure 1d). At the critical stress (G′ = G″), the viscoelastic network began to fracture and flow, thus resulting in liquid‐like behavior (G″ > G′). This allowed the ink to undergo continuous extrusion through micron‐sized nozzles. Rheological results show that the VCGQD nanocomposite gel demonstrated favorable shear‐thinning performance, high viscosity, rapid viscosity recovery, and other characteristics; thus, it can be used for extrusion‐based 3D printing. To further demonstrate the printability of the mixed hydrogels, various complex 3D structures were constructed by continuously extruding the nanocomposite gel without additives (Figure 1e–i). The printable rheological properties of the negative electrode materials significantly affected the construction of asymmetric MSCs. Therefore, the rheological and mechanical properties of MXene hydrogels were investigated. The characterization results showed that the MXene hydrogel possessed the same rheological and mechanical properties as the VCGQD hydrogel, which was conducive to the smooth extrusion of 3D printing (Figure S1, Supporting Information).

**Figure 2:**
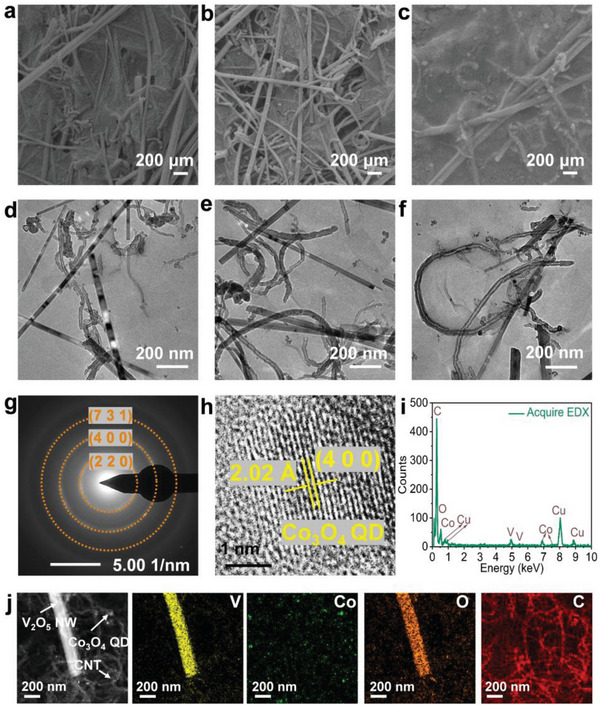
SEM of a) VCGQD‐1 mixed ink; b) VCGQD‐2 mixed ink; c) VCGQD‐3 mixed ink; TEM of d) VCGQD‐1 mixed ink; e) VCGQD‐2 mixed ink; f) VCGQD‐3 mixed ink; g) SAED; h) HRTEM image; i) EDS spectra; j) HAADF‐STEM images and elemental mapping of VCGQD‐2 mixed ink.

First, we synthesized Co_3_O_4_ QDs via a simple reflux method. By optimizing the mass ratio of Co_3_O_4_ QDs in mixed ink and mixing them with GO, CNT, and V_2_O_5_ NW dispersions, VCGQD‐based inks were successfully prepared for 3D printing. The microstructures of the prepared VCGQDs composite materials were characterized using scanning electron microscopy (SEM) and transmission electron microscopy (TEM). A comparison with SEM and TEM images of the sample without Co_3_O_4_ QDs show that the Co_3_O_4_ QDs were successfully inserted and maintained the integrity of the morphology (**Figure** **2**a–c; Figures S2–S4, Supporting Information). Meanwhile, the TEM images show that Co_3_O_4_ QDs coexisted with GO, CNTs, and V_2_O_5_ *NWs in the ink, and that the content increased gradually with the amount of Co_3_O_4_ QDs inserted, without significant aggregation (Figure 2d–f). The remaining porous structure promoted electrolyte permeation and ion diffusion. The TEM and SEM results showed good agreement. The electron diffraction pattern of the Co_3_O_4_ QDs in the ink showed diffraction rings corresponding to the (7 3 1), (4 0 0), and (2 2 0) crystal planes of Co_3_O_4_ (Figure 2g). Results of HRTEM show the exposure of Co_3_O_4_ (4 0 0) crystal planes in the ink (Figure 2h). SAED and HRTEM analyses of V_2_O_5_ NWs in the mixed hydrogel indicate no phase changes in the hydrogel (Figure S5, Supporting Information). Results of Energy dispersion spectroscopy (EDS) analysis show the presence of Co in the mixed ink (Figure 2i). In addition, high angle annular dark field scanning transmission electron microscopy (HAADF‐STEM) and the corresponding element mapping images confirmed the presence of V, Co, C, and O in the VCGQD‐2 mixed ink, thus further confirming the successful incorporation of Co_3_O_4_ QDs into the VCGQD‐2 mixed ink (Figure 2j).

**Figure 3:**
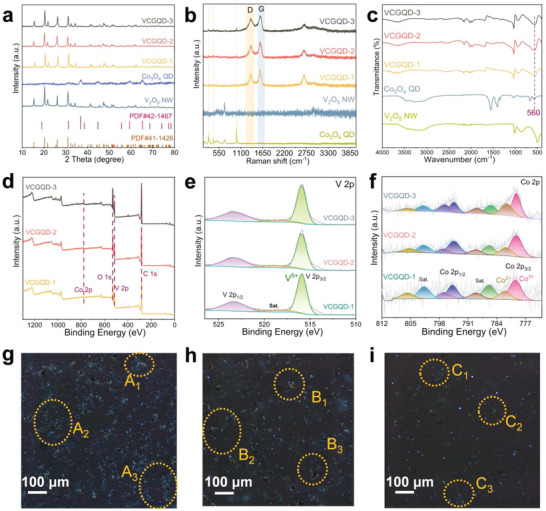
a) XRD; b) Raman; c) FT‐IR of all samples (such as:V_2_O_5_ NW,Co_3_O_4_ QD, VCGQD‐1 mixed ink, VCGQD‐2 mixed ink and VCGQD‐3 mixed ink); d) XPS full spectrum; e) V 2p XPS spectra; f) Co 2p XPS spectra of VCGQD‐1 mixed ink, VCGQD‐2 mixed ink, and VCGQD‐3 mixed ink; g) Dark field POM microscopic images of VCGQD‐1 mixed ink; h) Dark field POM microscopic images of VCGQD‐2 mixed ink; i) Dark field POM microscopic images of VCGQD‐3 mixed ink.

The phase structure characteristics of the VCGQD‐1, VCGQD‐2, and VCGQD‐3 mixed inks were analyzed using X‐ray diffraction (XRD), Raman spectroscopy, and Fourier transform infrared spectroscopy spectroscopy (FT‐IR) spectroscopy. Based on a comparison between the XRD spectra of pure individual materials and the standard spectrum (**Figure** **3**a; Figure S6, Supporting Information), there was no significant change in the XRD peak values of the mixed ink, indicating the phase structure of each component in the mixed ink did not change. However, owing to the low amount of Co_3_O_4_ QDs and the unsatisfactory crystal form, the XRD spectrum of the mixed ink did not show a clear Co_3_O_4_ peak. However, in the Raman spectrum (Figure 3b), three peak values similar to that of the Co_3_O_4_ QDs appeared in the mixed ink, thus confirming the successful addition of Co_3_O_4_ QDs. In addition, a metal oxygen bond peak similar to that of the Co_3_O_4_ QDs appeared at the 560 cm^−1^ peak position in the infrared spectrum (Figure 3c), thus further confirming the presence of Co_3_O_4_ QDs in the mixed ink. This is consistent with the results of electron diffraction and high‐resolution lattice fringe characterization. X‐ray photoelectron spectroscopy (XPS) can be used to characterize the valence states and existing forms of elements in various ink components. The full spectrum shows that Co 2p, O 1s, V 2p, and C 1s appeared in all three mixed inks (Figure 3d). In the high‐resolution V 2p spectrum (Figure 3e), the peak centered at 515.89 eV corresponds to the V^5+^. As shown in Figure 3f, for Co 2p, the two peaks at 779.5 and 783.3 eV can be attributed to Co^3+^ and Co^2+^ in the Co_3_O_4_ QDs, thus further proving that the state of the Co_3_O_4_ QDs in the ink remained unchanged. The conductivity of GO was further improved by reducing the hydrogel electrode with hydrazine hydrate. The structure of the reduced ink was further analyzed via XRD, Raman, FT‐IR spectroscopy, and XPS. Figure S7 (Supporting Information) shows that there is no significant change in the peak position of the ink phase after reduction. The strength ratio of *I_D_* to *I_G_* can further describe the graphitization degree of the material. A comparison of the *I_D_/I_G_* values calculated using Raman spectroscopy before and after reduction shows that the *I_D_/I_G_* values of the ink after reduction (1.0629, 0.8601, and 0.9950) were higher than those before reduction (0.8182, 0.8327, and 0.7101) (Figure S8, Supporting Information).^[^ ^41^, ^42^ ^]^ A lower graphitization rate implies more defects, and larger defects increase the polarization loss, thereby affecting the electrochemical performance. After the reduction, the peak values of the IR spectra changed (Figure S9, Supporting Information). Based on a comparison with the infrared spectra of the Co_3_O_4_ QDs and V_2_O_5_ NWs, the peak values at 478 and 630 cm^−1^ correspond to V─O and Co─O metal oxygen bonds, respectively. Meanwhile, a comparison of the XPS spectra shows that only the valence state of V changed before and after reduction (Figures S10 and S11, Supporting Information), and a low valence state V(V^4+^) appeared.

**Figure 4:**
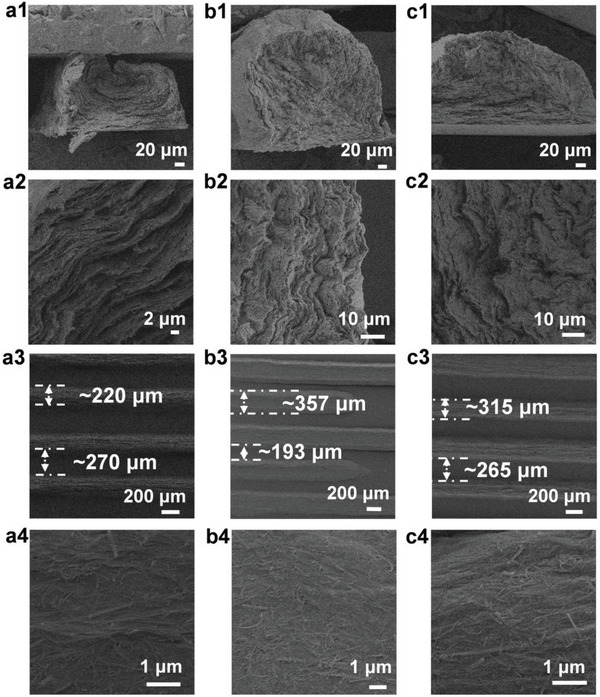
SEM image of electrode cross‐section: a1) VCGQD‐1; b1) VCGQD‐2; c1) VCGQD‐3; Magnified SEM image of electrode cross‐section: a2) VCGQD‐1; b2) VCGQD‐2; c2) VCGQD‐3; Top‐down SEM image of electrode: a3) VCGQD‐1; b3) VCGQD‐2; c3) VCGQD‐3; Magnified top‐down SEM image of electrode: a4) VCGQD‐1; b4) VCGQD‐2; c4) VCGQD‐3.

The phase behavior changes in the ink after the addition of Co_3_O_4_ QDs were investigated using a polarization optical microscope (POM). The occurrence of birefringence between the polarizers is a direct evidence of solution‐induced liquid‐crystal phase formation. Birefringence was observed in the ink with different proportions of Co_3_O_4_ QD intercalation, which indicates a change in the isotropic nematic phase (I‐N). A comparison of the results obtained via light‐ and dark‐field microscopy shows that the ink embedded with different proportions of Co_3_O_4_ QDs exhibited stable birefringence (Figure 3g–i; Figures S12 and S13, Supporting Information), diffused throughout the dispersion, and exhibited a dynamic schlieren texture as well as other nematic features. The presence of schlieren texture means that the insertion of Co_3_O_4_ QDs does not affect the long‐range orientation of the ink mixture. A layered ordered electrode structure was constructed using the characteristics of long‐range ordered orientation of materials and the shear force field provided by 3D printing technology. The presence of the schlieren texture indicates that the insertion of Co_3_O_4_ QDs does not affect the long‐range orientation of the ink mixture. A layered ordered electrode structure was constructed by considering the long‐range ordered orientation of materials and the shear force field provided by 3D printing technology. After verifying the liquid‐crystal characteristics of the different Co_3_O_4_ QD intercalation inks, the microstructures and morphology of the 3D printed interdigital electrodes were investigated. The cross‐section and surface of the 3D printed electrodes with different proportions of Co_3_O_4_ QDs embedded ink were observed via SEM. The cross‐section of each electrode was stacked in a layered and orderly manner (**Figure** **4**a1–c2; Figure S14, Supporting Information), and as the amount of Co_3_O_4_ QD intercalation increased gradually, the layered structure became denser. In addition, the top‐down SEM images show that the mixed materials inside the printed electrode exhibited a certain orientation(Figure 4a4–c4). The finger width and interdigital width can affect the transport of charged ions during electrochemical reactions. A smaller interdigital width is conducive to the rapid transfer of electronic ions. Therefore, optical images of MSCs printed with a nanocomposite gel featuring different amounts Co_3_O_4_ QDs were analyzed. The resolution of the MSC electrodes printed using the VCGQD‐2 nanocomposite gel was much higher than those printed using VCGQD‐1 and VCGQD‐3 (Figure 4a3–c3). VCGQD electrodes printed with uniform edge structures and digital widths can effectively reduce the possibility of short circuits.

To further analyze the effect of the intercalation of the 0D dot structure on the liquid‐crystal characteristics of the mixed gel and the 3D printing of the electrode, we added 0D CDs to GO, CNT, and V_2_O_5_ NWs dispersions. The XRD spectra before and after the reduction show that the 0D CDs did not affect the phase structure of the material (Figures S15 and S16, Supporting Information). Meanwhile, an investigation into the rheological mechanics of the VCGCD mixed gel shows that the gel exhibited printable rheological mechanical properties similar to those of the VCGQD mixed gel (Figure S17, Supporting Information). SEM and TEM images show that the uniform distribution of CDs in the ink was beneficial to the uniform distribution of CDs in the printing electrode (Figures S18 and S19, Supporting Information). In addition, the characterization of the internal structure of the printed electrode embedded with CDs shows that the embedding of CDs did not affect the ordered liquid‐crystal characteristics of the mixed gel or the formation of the layered structure (Figure S20, Supporting Information).

**Figure 5:**
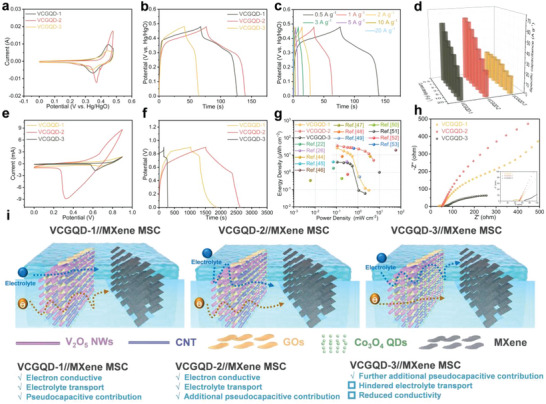
The electrochemical performance of the three electrodes: a) CV curves of each electrode at a sweep of 50 mV s^−1^; b) The GCD curves of each electrode were compared under the current density condition of 0.5 A g^−1^; c) GCD curves of VCGQD‐2 electrodes under different current density; d) The comparison diagram of specific capacitance of each sample at different current densities; 3D printed electrode VCGQD//MXene MSCs electrochemical performance: e) When the scanning speed is 50 mV s^−1^, compare the CV curves of each MSCs; f) The GCD curves of all MSCs devices are compared at a current density of 0.48 mA cm^−2^; g) Ragone plot; h) EIS spectra for all electrodes; i) Schematic of electrolyte/electron transport path in VCGQDs//MXene MSCs.

First, we evaluated the electrochemical performances of VCGQD‐1, VCGQD‐2, and VCGQD‐3 in aqueous two‐ and three‐electrode systems. The cyclic voltammetry (CV) and galvanostatic charge discharge (GCD) curves of the three electrodes showed that these samples presented typical redox peaks of battery‐type batteries. The CV curves in **Figure** **5**a show that the redox current of VCGQD‐2 was much higher than those of the VCGQD‐1 and VCGQD‐3 electrodes. The electrochemical reaction kinetics of the electrode preparation were investigated at different scanning rates (Figures S21–S26, Supporting Information). As the scanning rate increased, the reduction peak (*peak 1*) shifted toward a positive voltage, whereas the oxidation peak (*peak 2*) shifted toward a negative voltage. This may be due to an increase in the internal diffusion resistance at high scanning rates. In addition, at high scanning rates, the redox peaks of VCGQD‐1, VCGQD‐2, and VCGQD‐3 remained unchanged, thereby indicating their excellent capacitance and rate capability. The current *(i)* and scanning rate *(v)* are correlated as *i* = av*^b^* (see Supporting Information for details).^[^ ^43^ ^]^ The value of *b* ranged from 0.5 to 0.7, thus indicating simultaneous diffusion control and surface capacitance control processes in the entire electrochemical reaction. Subsequently, the contribution ratios of the two capacitance mechanisms were calculated at different scanning rates. The calculation results show that, as the scanning rate increased, the contribution rate of the capacitance increased gradually, thus indicating efficient charge storage. As shown in Figures S21c, 23c, and S25c (Supporting Information), VCGQD‐2 had a higher diffusion‐controlled contribution than the other electrodes, which indicates that the corresponding electrochemical reactions in the VCGQD‐2 electrodes were mainly diffusion controlled. The electrochemical behavior of VCGQD‐1, VCGQD‐2, and VCGQD‐3 was further investigated using GCD experiments. Based on Figure 5b, the GCD curve of 0.5 A g^−1^ proves that the VCGQD‐2 electrode had a longer discharge time compared with the other electrodes. Meanwhile, the GCD curve of the VCGQD‐2 electrode at different current densities (see Figure 5c,d; Figure S27, Supporting Information) indicates that the VCGQD‐2 electrode had the longest charging and discharging times and the highest specific capacitance at a current density of 0.5 A g^−1^.

Additionally, an aqueous asymmetric supercapacitor (ASC) device was constructed using VCGQDs (positive) and MXenes (negative) using a previously reported method.^[^ ^27^ ^]^ As shown in Figures S28–S34 (Supporting Information), the CV curve shape of the VCGQD//MXene ASC devices can remained unchanged at different scanning rates, thus exhibiting the typical pseudo‐capacitive performance and fast reversible charge storage capability. CV tests at different scanning rates further confirmed the interface capacitance behavior of all the VCGQD//MXene ASC devices. By calculating and plotting the relationship between log *(i)* and log *(v)*, we discovered that the b values of the VCGQD‐1, VCGQD‐2, and VCGQD‐3//MXene ASC devices ranged from 0.5 to 1. This indicates that the overall charge storage of the VCGQD‐1, VCGQD‐2, and VCGQD‐3//MXene ASC devices was mainly controlled by the surface capacitors. Based on the calculated capacitance contribution rate, the contribution of surface capacitance control was more significant in the VCGQD‐1//MXene ASC devices. As the amount of Co_3_O_4_ QDs increased, the surface capacitance control ratio decreased gradually, and the diffusion control process intensified gradually. This may have been limited by the gradual densification of the structure inside the electrode as the number of inserted QDs increased gradually. The surface capacitance control contribution rate of VCGQD‐2//MXene increased gradually from 34% to 69% as the scanning rate increased, thus indicating its favorable charge‐transfer kinetics. At 50 mV s^−1^, VCGQD‐2//MXene constituted 61% of the shadow area controlled by surface capacitance. The GCD curves of the VCGQD‐1, VCGQD‐2, and VCGQD‐3//MXene ASC devices assembled at different current densities were obtained. At a current density of 0.5 A g^−1^ (see Figures S28b and S35, Supporting Information), the specific capacitance of the VCGQD‐2//MXene ASC device was 18.7 mF cm^−2^, which exceeded those of the other devices. This is mainly because the addition of an appropriate amount of Co_3_O_4_ QDs appropriately increased the pseudo‐capacitance while simultaneously maintaining a certain spatial structure, which was conducive to the immersion of the electrolyte and the transfer of electronic charges.

KOH was dispersed in the polyvinyl alcohol (PVA) gel solution to create the electrolyte, VCGQDs were used to construct the cathode using a 3D printed hydrogel electrode, a 3D printed MXene hydrogel was used as the negative electrode, and flexible polyethylene terephthalate (PET) was used as the substrate to prepare an asymmetric MSC. The clear redox peaks in the CV curves of the 3D‐printed MSCs devices at different scanning rates confirmed the Faraday reaction behavior of all the 3D printed MSCs (Figure S36, Supporting Information). The CV curve shape was retained at different scanning speeds, thus demonstrating the excellent rate capacity and redox capacitance. The peak current of the VCGQD‐2//MXene MSC significantly exceeded those of the VCGQD‐1//MXene MSC and VCGQD‐3//MXene MSC devices at a scanning rate of 50 mV s^−1^, thus indicating the better electrochemical activity of the VCGQD‐2//MXene MSC (Figure 5e). Based on a comparison with the GCD curve at a current density of 0.48 mA cm^−2^, the discharge time of VCGQD‐2//MXene was significantly longer than those of VCGQD‐1//MXene and VCGQD‐3//Mxene (Figure 5f; Figures S37 and S38, Supporting Information). Under high current densities, the charge–discharge curve of the VCGQD‐2//MXene MSC remained unchanged. This indicates that their electrochemical properties are superior to those of recently reported materials (Table S1, Supporting Information). Compared with the other two types of MSCs and the addition of non‐metallic CDs (Figure S39, Supporting Information), the VCGQD‐2//MXene MSC exhibited better electrochemical performance, which may be due to the appropriate improvement in capacitance characteristics via the further addition of Co_3_O_4_ QDs. Electrochemical tests were conducted on the Co_3_O_4_ QDs and CDs aqueous electrodes, and the CV and GCD curves of Co_3_O_4_ QDs showed that the electrochemical performance of Co_3_O_4_ QDs is better than that of CDs. (Figures S40 and S41, Supporting Information) Meanwhile, by controlling the amount of Co_3_O_4_ QDs in the layered structure, the compactness of the layered structure can be adjusted to adjust the charge and ion transport paths between the electrodes, thereby further improving the conductivity of the MSCs. (Figure 5i) This is consistent with the results of the VCGQD‐3//MXene MSC with a high impedance, as shown by the electrochemical impedance spectroscopy (EIS) result (Figure 5h). The area energy and power densities of the microenergy‐storage devices were compared. Conventional SCs are typically compared based on their unit mass. A Ragone diagram of the area energy density and power density of the 3D printed MSC from this study was compared with those of the MSCs constructed in other studies, as shown in Figure 5g.^[^ ^22^, ^26^, ^44^, ^45^, ^46^, ^47^, ^48^, ^49^, ^50^, ^51^, ^52^, ^53^ ^]^ The excellent electrochemical performance of the 3D printed VCGQD/MXene MSCs can be attributed to the insertion of an appropriate amount of active material into the layered structure, thus resulting in high‐quality loading, a large capacity, a suitable specific surface area, and a controllable loose‐layered macroporous structure (Figure S42, Supporting Information). In addition, Co_3_O_4_ QD intercalation in the 3D‐printed layered electrode structure and the uniform distribution can further eliminate electrochemical surface effects on the printed electrode. The coordination between the layered structures of the printed positive and negative electrodes further promotes the transfer and transmission of charges between the layered structures of the positive and negative electrodes (Figure S43, Supporting Information).

The advantages of the VCGQD‐2 gel over the VCGQD‐1 and VCGQD‐3 gels as cathode materials were analyzed via in‐situ XRD by analyzing the phase change of the VCGQD//MXene MSCs during the charging and discharging processes. The changes in the (2 2 0), (3 1 1), (4 0 0), (5 1 1), and (4 0 0) crystal planes of the Co_3_O_4_ QDs and the (2 0 0) crystal planes of the V_2_O_5_ NWs during the electrochemical charge–discharge reaction of the mixed gel were analyzed based on the peak position changes of the active substances. During the reaction, the peak positions of the MSCs shifted, but the intensities and positions of the peaks before and after the reaction remained unchanged, which implies that the reversible phase did not change significantly during the charging and discharging processes (Figure S44, Supporting Information). Based on the charge–discharge curve, the (2 0 0) crystal planes of the V_2_O_5_ NWs and the Co_3_O_4_ QDs of the VCGQD‐2//MXene MSCs reacted more rapidly during the charging process compared with those of the other two MSCs and ultimately recovered to the original peak position, thereby further proving the rapid transfer of charge in the VCGQD‐2//MXene MSCs during the reaction process. This is conducive to the rapid progress of the electrochemical reactions and improved the performance.

## 3 Conclusion

In summary, nanocomposite inks were prepared via extrusion 3D printing using Co_3_O_4_ QDs, GO, CNTs, and V_2_O_5_ NWs to be used as high‐resolution interdigital electrodes. An ink with liquid‐crystal properties was successfully constructed into the internal layer of an electrode. CNT and GO as conductive agents and binders can effectively improve the conductivity of MSCs electrodes and further enhance their electrochemical performance. V_2_O_5_ NWs active materials with liquid crystal properties are conducive to the formation of controllable layered structures. The addition of 0D Co_3_O_4_ QDs with pseudocapacitive properties can effectively reduce the impact of surface or near surface on electrochemical performance by inserting them into interconnected porous structures without changing the printable rheological properties, which is conducive to further improving the electrochemical performance of electrodes. The effects of Co_3_O_4_ QDs and CDs on the liquid‐crystal properties, electrode structure, rheological mechanics, and electrochemical performance of the mixed ink were investigated. The pore structure of the layered electrode was adjusted by varying the intercalation amount of Co_3_O_4_ QDs, and the surface pores and oxidation active sites synergistically optimized the electrode structure and further improved the electrochemical performance. In addition, compared with the performance of other 0D non‐metallic oxide carbon electrodes, the layered structure of the Co_3_O_4_ QD‐inserted electrode exhibited superior electrochemical activity. This can be attributed to the addition of the Co_3_O_4_ QD pseudocapacitive material, which improved the capacitance. Moreover, by controlling the insertion amount of Co_3_O_4_ QDs in the layered structure and regulating the density of the layered structure, the conductivity of the MSCs improved because of the increase in charges and the ion transport between the electrodes. In addition, the intercalation of QDs allows the pore structure of the electrode surface to be adjusted, thereby reducing the adverse effects of the surface and near‐surface mechanisms on the capacitance and energy density of the MSCs and effectively improving their electrochemical performance. In situ XRD analysis was conducted to understand the superiority of VCGQD‐2 over the other MSCs in terms of electrochemical performance. The results confirmed the significance of material properties, electrode structure construction, and material intercalation in the development of EESE. This study provides new research directions and guidance for the development of next‐generation high‐performance 3D printed energy materials, the construction of device structures, and the adjustment and improvement of performance.

## Conflict of Interest

The authors declare no conflict of interest.

## Supporting information

Supporting Information

## Data Availability

Research data are not shared.
