# Supplementary material for: Co3O4 Quantum Dots Intercalation Liquid‐Crystal Ordered‐Layered‐Structure Optimizing the Performance of 3D‐Printing Micro‐Supercapacitors
Source: Adv Sci (Weinh). 2023 Sep 26;10(33):2303636. doi: 10.1002/advs.202303636 (PMC10667828; doi:10.1002/advs.202303636)
Supplement: Supplementary file 1 — Supporting Information [file ADVS-10-2303636-s001.pdf]

ADVANCED SCIENCE
Open Access

Supporting Information

for *Adv. Sci.*, DOI 10.1002/advs.202303636

Co_3O_4 Quantum Dots Intercalation Liquid-Crystal Ordered-Layered-Structure Optimizing the Performance of 3D-Printing Micro-Supercapacitors

Huijie Zhou, Yangyang Sun, Hui Yang, Yijian Tang, Yiyao Lu, Zhen Zhou, Shuai Cao, Songtao Zhang, Songqing Chen, Yizhou Zhang and Huan Pang**

Supporting Information

Co_3O_4 Quantum Dots Intercalation Liquid-crystal Ordered-layered-structure optimizing the Performance of 3D-printing Micro-supercapacitors

*Huijie Zhou, Yangyang Sun, Hui Yang, Yijian Tang, Yiyao Lu, Zhen Zhou, Shuai Cao, Songtao Zhang, Songqing Chen and Huan Pang**

H. Zhou, Y. Sun, H, Yang, Y. Tang, Y. Lu, Z. Zhou, S. Cao, S. Zhang, S.Chen, H. Pang
School of Chemistry and Chemical Engineering, Yangzhou University, Yangzhou, Jiangsu, 225009, P. R. China
E-mail: huanpangchem@hotmail.com (H. Pang); panghuan@yzu.edu.cn

Content

1. Experimental Procedures 3
2. Calculations 5
3. Rheological mechanics test of MXene hydrogel 6
4. SEM and TEM of Co_3O_4 QDs 7
5. TEM of VCGQD-2 8
6. SEN and TEM diagrams of mixed hydrogel before undoped Co_3O_4 QDs. 8
7. HRTEM and SAED spectra of V_2O_5 NWs in the mixed hydrogel 9
8. XRD of Co_3O_4 QDs 9
9. After reduction: XRD of all mixed gel 10
10. After reduction: Raman 11
11. After reduction: FT-IR 12
12. Before reduction: XPS spectra of the 13
13. After reduction: XPS of all mixed gel 14
14. Polarization microscope spectrogram under bright field conditions 15
15. Polarization microscope spectrogram of VCG and VCGCDs 16
16. SEM image of VCG electrode cross-section 17
17. Before reduction: XRD of VCGCDs 18
18. After reduction: XRD of VCGCDs 19
19. Rheological mechanics test of VCGCDs hydrogel 20
20. TEM of VCGCDs mixed hydrogel 21
21. SEM of VCGCDs mixed hydrogel 22
22. SEM image of VCGCDs electrode cross-section 22
23. The analysis of ion-diffusion and capacitive contributions of the VCGQD-1 23
24. Pseudocapacitive contribution shadow diagram of VCGQD-1 in CV curves 24
25. The analysis of ion-diffusion and capacitive contributions of the VCGQD-2 25
26. Pseudocapacitive contribution shadow diagram of VCGQD-2 in CV curves 26
27. The analysis of ion-diffusion and capacitive contributions of the VCGQD-3 27
28. Pseudocapacitive contribution shadow diagram of VCGQD-3 in CV curves 28
29. Three-electrode systems: the GCD curves of VCGQD-1 and VCGQD-3 at different current densities 29
30. CV and GCD curves of the VCGQD//MXene at sample potentials and scan rate 29
31. The analysis of ion-diffusion and capacitive contributions of the VCGQD-1//MXene 30
32. Pseudocapacitive contribution shadow diagram of VCGQD-1//MXene in CV curves 31
33. The analysis of ion-diffusion and capacitive contributions of the VCGQD-2//MXene 32
34. Pseudocapacitive contribution shadow diagram of VCGQD-2//MXene in CV curves 33
35. The analysis of ion-diffusion and capacitive contributions of the VCGQD-3//MXene 34
36. Pseudocapacitive contribution shadow diagram of VCGQD-3//MXene in CV curves 35
37. CV and GCD curves of the VCGQD-1//MXene at different potentials 36
38. CV curves of the VCGQD-1//Mxene and VCGQD-3//Mxene MSCs at scan rates 37
39. GCD curves of the VCGQD-1//Mxene and VCGQD-3//Mxene MSCs at different current density 38
40. Electrochemical characterization of the VCGQD-2//MXene MSC 39
41. CV and GCD curves of the VCGCDs//MXene 40
42. CV and GCD curves of the Co_3O_4 QDs 41
43. CV and GCD curves of the CDs 41
44. BET and pore size distribution 42
45. SEM image of VCGCDs electrode cross-section 43
46. In situ XRD spectra during charging and discharging 44
Tabel S1 45

1. Experimental Procedures

Materials and instruments

V_2O_5 powder, Cobalt acetate($C_4H_6CoO_4$), Benzylalcohol (C_7H_8O), ammonium hydroxide (NH_4OH), polyvinyl alcohol powder (PVA), KOH, CNT, GO, HCl and HF were bought from Aladdin Industrial Corporation. Titanium Aluminum Carbide (Ti_3AlC_2) was purchased from Laizhou Kai Kai Ceramic Materials Co., Ltd.

The microstructures and morphology of samples were observed by field emission scanning electron microscopy (FE-SEM, ZeissSupra55) under the acceleration voltage of 5.0 kV and transmission electron microscopy (TEM, JEM-2100 instrument). High-resolution TEM (HRTEM) images, selected area electron diffraction (SAED) images, and elemental mapping were captured on a Tecnai G2 F30 at an acceleration voltage of 300 kV. The crystal phase was performed by X-ray diffraction (XRD) on a Bruker D8 Advanced X-ray Diffractometer (Cu-Kα radiation: λ = 0.15406 nm). Fourier transform infrared (FT-IR) spectra were captured on TENSOR27. Raman spectra were carried out on INVIA REFLEX. The X-ray photoelectron spectra (XPS) was obtained on a Thermo Scientific ESCALAB 250 apparatus. The value of specific surface area was calculated using the Brunauer-Emmett-Teller (BET) method (ASAP 2469 model). The pore size was obtained from the adsorption-desorption branch of the nitrogen isotherms by the Barrett-Joyner-Halenda method.

Synthesis of V_2O_5 NWs

0.36 g of V_2O_5 powder was dispersed in 30 ml of deionized water. Then, 10 ml 30% hydrogen peroxide was added, and stirred at room temperature for 2 h, transferred to Teflon lined sealed autoclave at 200 °C for 96 h. V_2O_5 NWs were prepared by natural cooling, deionized water washing for 6 times and freeze-drying.

Synthesis of Co_3O_4 QD

200 mg of cobalt acetate was dispersed in 10 ml of benzyl alcohol solvent and stirred continuously at room temperature for 1 hour. Subsequently, 8 ml of ammonium hydroxide solution was added and stirred vigorously for 10 minutes. The solution turned reddish brown. Then stir at 170 ℃ for 4 hours. As the volume gradually decreases, an inky black suspension is finally obtained. An appropriate amount of ether solvent is added to the reaction solution and centrifuged to collect the black precipitate. Before characterization, the sample is washed with ethanol and dried.

Synthesis of $Ti_3C_2T_X$ nanosheets

$Ti_3C_2T_X$ nanosheets was prepared in a typical method. 1 g Ti_3AlC_2 was added to the etchant solution, which was consist of 6 mL HCl, 1 mL HF and 3 mL deionized water (DI water). The reaction was stirred at 400 rpm for 15 h at 41 °C. Then, the resultant was washed with DI water repeatedly until pH is neutral (4500 rpm, 5 min) and the accordion-liked $Ti_3C_2T_X$ was obtained. Subsequently, 25 mL DI water containing 1.5 g of LiCl was added. After reaction for 2 h, the resultant was washed with DI water repeatedly (3500 rpm, 5 min) and delaminated manually by hand shaking agitation and centrifugation to obtain Ti3C2TX suspension.

Preparation of VCGQD-1, VCGQD-2, VCGQD-3 hydrogels

10 mg 8.5 mg ml^{-1} graphene oxide dispersion (Tanfeng Tech.Inc.) and 45 mg CNTs (XFNANO, 14 wt%) dispersion were mixed. Then, 45 mg V_2O_5 NW and 2 mg Co_3O_4 QD were dispersed in above mixture. After ultrasonic for 60 min, the sequence of stirring for 60 min was repeated for many times until the mixture was uniform, the mixture was vacuum filtered through a polytetrafluoroethylene (PTFE) membrane filter with a pore diameter of 0.45 μm. Subsequently, the ink on the membrane filter was collected and stirred to obtain VCGQD-1 gel. VCGQD-2(4 mg Co_3O_4 QD) and VCGQD-3 (6 mg Co_3O_4 QD) were prepared by the same process.

Preparation of the Gel Electrolyte: Add 4.56 g polyvinyl alcohol (Xilong, PVA-124, AR) powder (PVA)was added to 30 ml deionized water and soaked, and then was heat to 80 °C until the solution becomes transparent. Meanwhile, 6.39 g KOH (Shanghai Aladdin Biochemical Technology Co., Ltd.) was dissolved in 15 ml deionized water. Afterwards the KOH solution was then slowly added dropwise to the PVA solution until the transparent gel state was achieved. After boiling, the 20 min bubbles were discharged. The final solution was cooled down to room temperature and dropped on the interphalangeal region of the printed MSCs to fully wet the electrode.

Preparation of 3D Printing MSCs: Firstly, according to the gelation strategy of modified nanocomposites, VCGQD electrodes and MXene electrodes for extrusion 3D printing is prepared. 1D CNT in ink is used as fluid collector and conductive agent to form interconnected conductive network. 2D GO nanosheet is used as adhesive. VCGQD hydrogels were prepared by Co_3O_4 QD, V_2O_5 NWs, CNTs and GO via micro ultrasonic and vacuum filtration. After the gels formulation were completed, they were deposited layer by layer on the polyethylene terephthalate (PET), and thick interdigital electrodes were constructed for MSCs using extrusion 3D printing. Then dry, remove the solvent, solidify the architecture of 3D printing, and use hydrazine hydrate vapor reduction process to reduce graphene oxide (rGO) for 12 h. Finally, the PVA-KOH gel electrolyte was dripped into the projection area of the microelectrode, and the directional network was completely filled in to finish the preparation of MSCs.

According to the formula, the ink was loaded into a 5 ml syringe barrel, then transferred into a 50 ml centrifuge tube, and centrifuged at 2500 rpm for 3 min to remove the internal bubbles before printing. 3D printing was performed using a desktop robot (XM-331) according to a pre-programmed program. The inner diameter of the passing needle was 210–230 μm. The ink is extruded by air pressure. The optimum extrusion pressure is 30–60 psi. The movement speed of the nozzle is 5–7 mm s^{-1}. First of all, 3D printing technology was used to print the designed pattern and electrode gel onto the PET substrate. The preset line spacing is 200–400 μm. The nozzle height is maintained at 200-300 μm or so. Then PVA-KOH gel electrolyte was used to cover the whole MSCs. Finally, the additional PET layer is used as a passivation layer to cover the gel electrolyte to enhance stability.

2. Calculations

The mass-specific capacitance (C/F g^{-1}) of the device can also be calculated using：

$$C=Q / (m\times V)= \int Idt / (m\times \triangle V)= I\times t_{discharge} / (m\times \triangle V) \quad (1)$$

where m is the mass of the activated materials, I is the discharge current, $t_{discharge}$ is discharge time, and $\triangle V$ is the potential drop during discharge.

The area-specific capacitance (C/mF cm^{-2}) of the device can also be calculated using：

$$C=Q / (A\times \triangle V)= \int Idt / (A\times \triangle V)= I\times t_{discharge} / (A\times \triangle V) \quad (2)$$

where A is the surface area of the device, I is the discharge current, $t_{discharge}$ is discharge time, and $\triangle V$ is the potential drop during discharge.

The kinetic of capacitive contribution can be obtained by calculating the CV curves at different scan rates. The relationship between current (i) and scan rate (v) can be written as:

$$i=av^b \quad (5)$$

$$log(i)=b\times log(v)+log(a) \quad (6)$$

where a and b are constant that can be obtained from log(v) versus log(i) plots. The situation where b=0.5 represents an ideal diffusion-controlled process and when b=1.0 indicates a surface capacitive-controlled process. The capacitive contributions at different scan rates can be calculated by the equations described as below:

$$i=k_1v+k_2v^{1/2} \quad (7)$$

$$i/v^{1/2}=k_1v^{1/2}+k_2 \quad (8)$$

where i is the current density at a voltage (V), v is the scan rate (mV s^{-1}), k_1 and k_2 can be obtained from the slope and intercept, respectively. Where k1v can be attributed to the current from surface capacitance contribution, while $k_2v^{1/2}$ is indexed to the diffusion process.

3. Rheological mechanics test of MXene hydrogel.

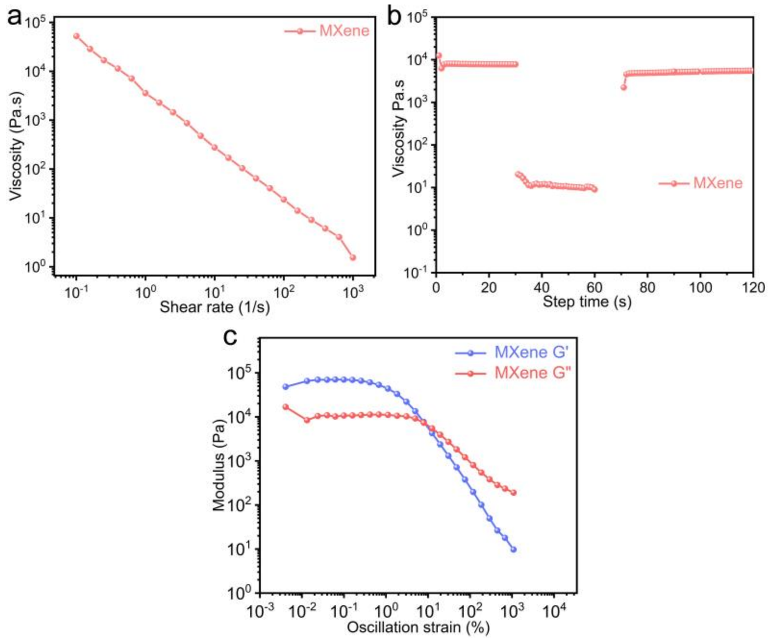

Figure S1. a) Apparent viscosity of the MXene hydrogels as a function of shear rate; b) PHS experiment; c) The G′ and G″ of MXene hydrogels.

4. SEM and TEM of Co_3O_4 QDs.

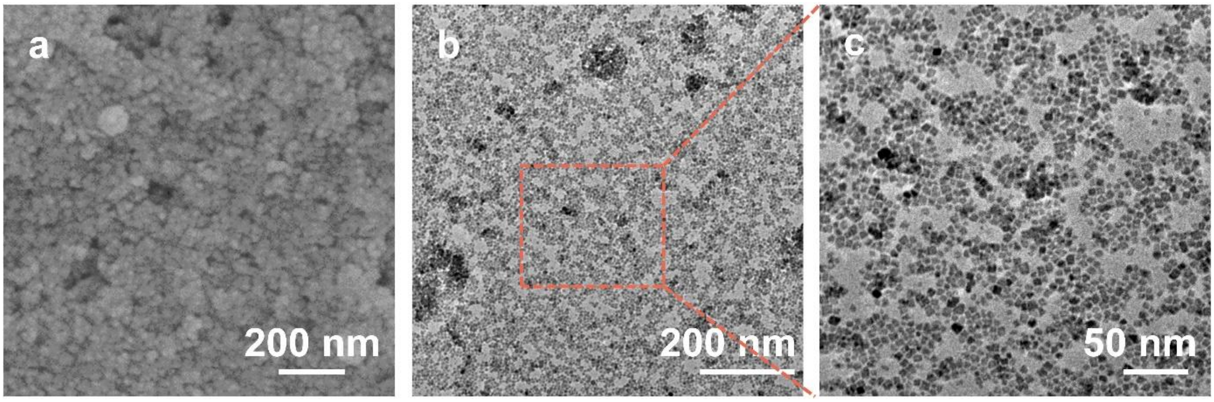

Figure S2. a) SEM; b, c)TEM of Co_3O_4 QDs.

5. TEM of VCGQD-2

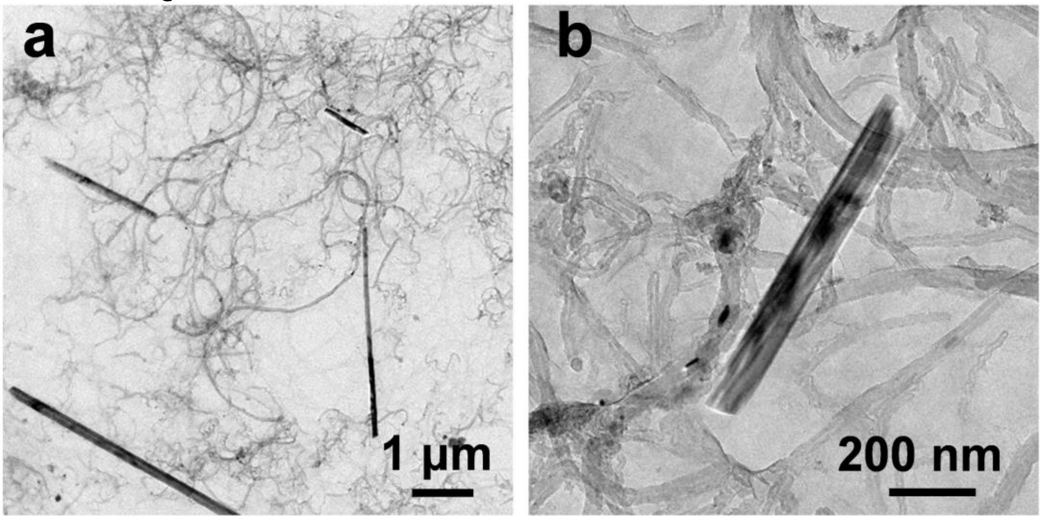

Figure S3. a,b) TEM of VCGQD-2.

6. SEN and TEM diagrams of mixed hydrogel before undoped Co_3O_4 QDs.

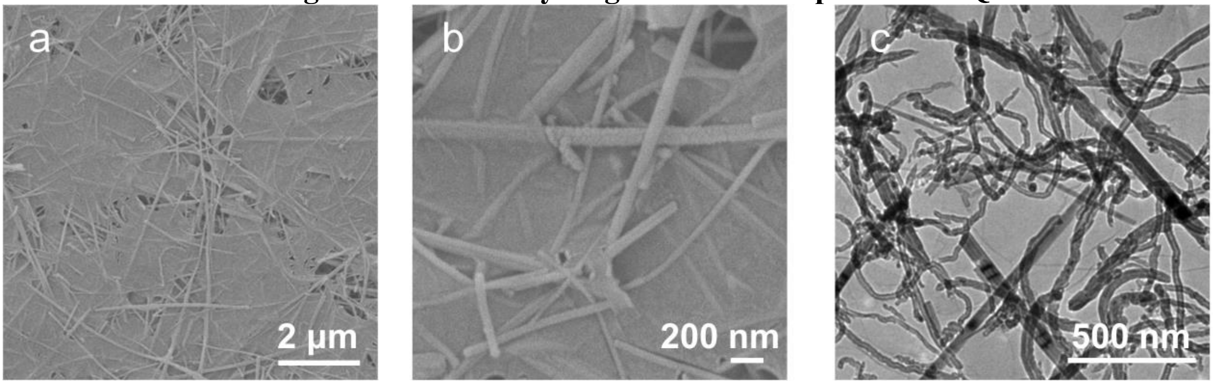

Figure S4. a, b) SEN and c) TEM diagrams of mixed hydrogel before undoped Co_3O_4 QDs.

7. HRTEM and SAED spectra of V_2O_5 NWs in the mixed hydrogel

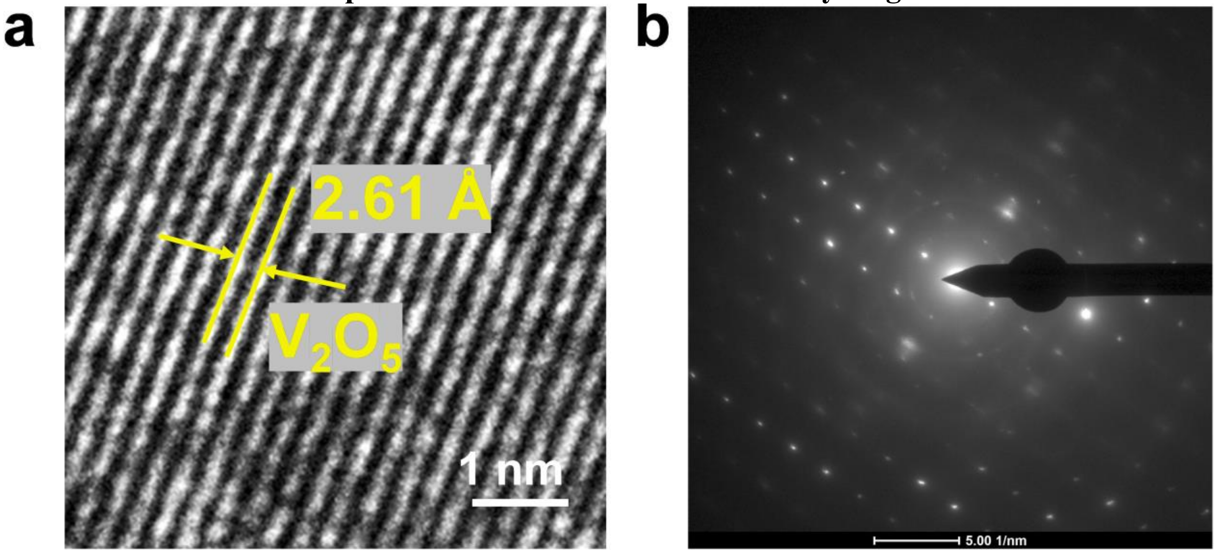

Figure S5. a) HRTEM and b) SAED spectra of V_2O_5 NW in the mixed hydrogel.

8. XRD of Co_3O_4 QDs

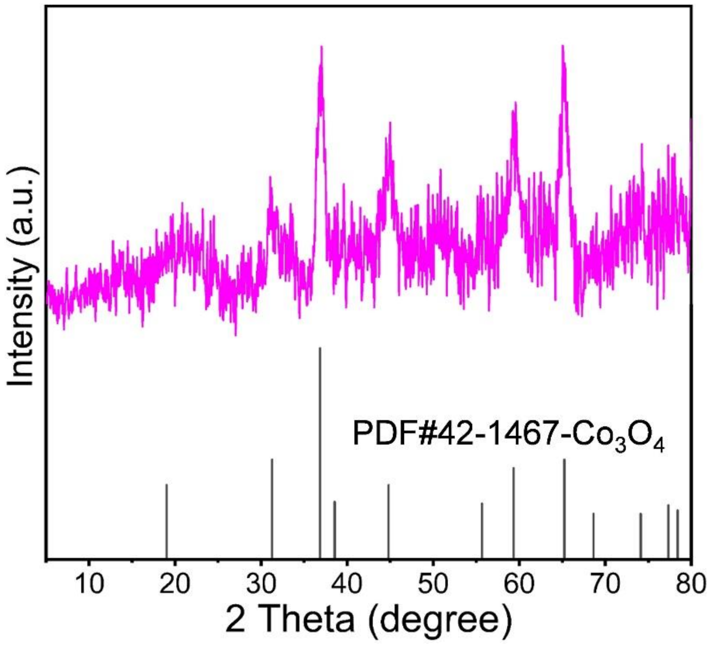

Figure S6. XRD of Co_3O_4 QDs.

9. After reduction: XRD of all mixed gel

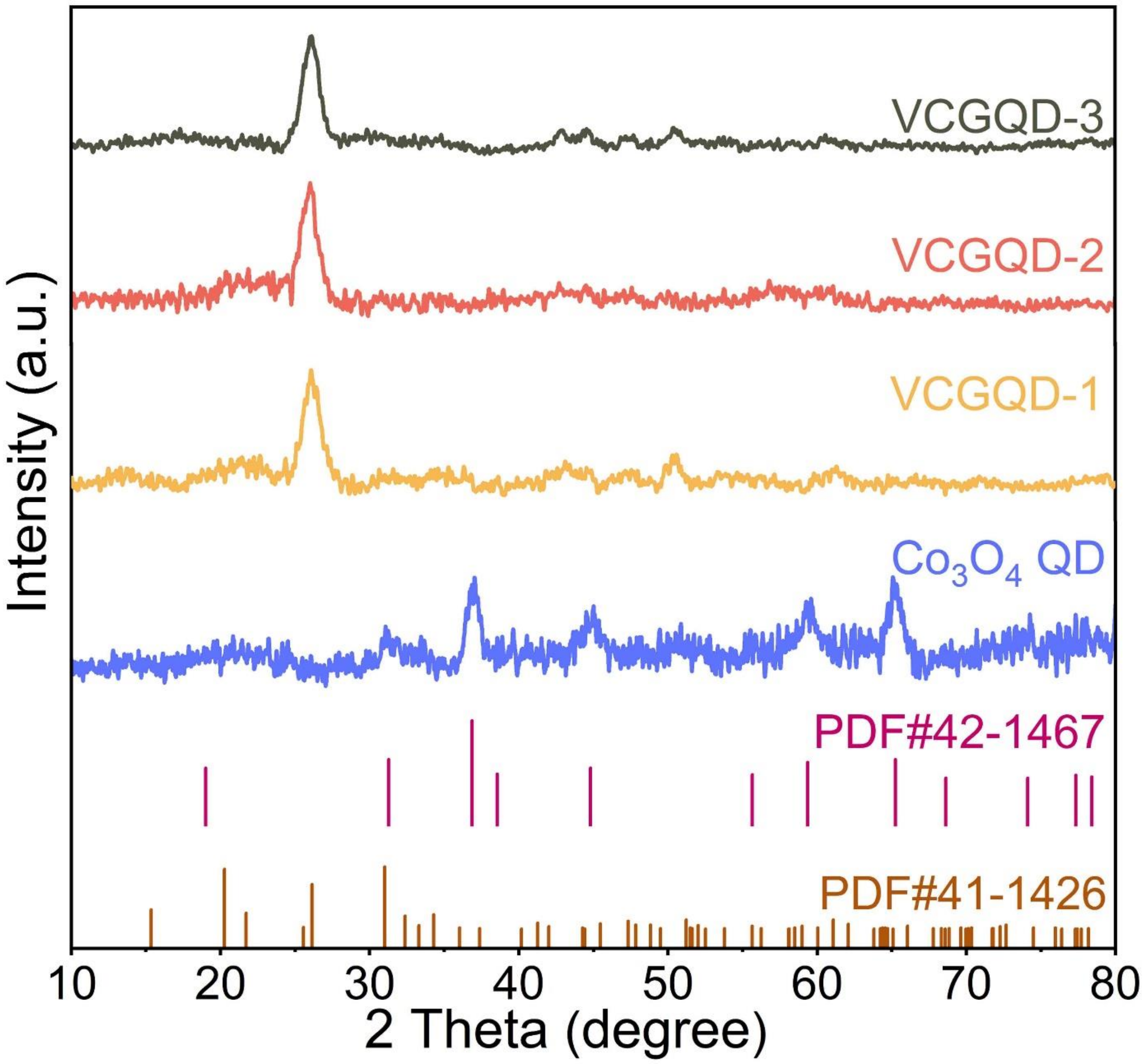

Figure 7. XRD patterns of all mixed gel after reduction.

10. After reduction: Raman

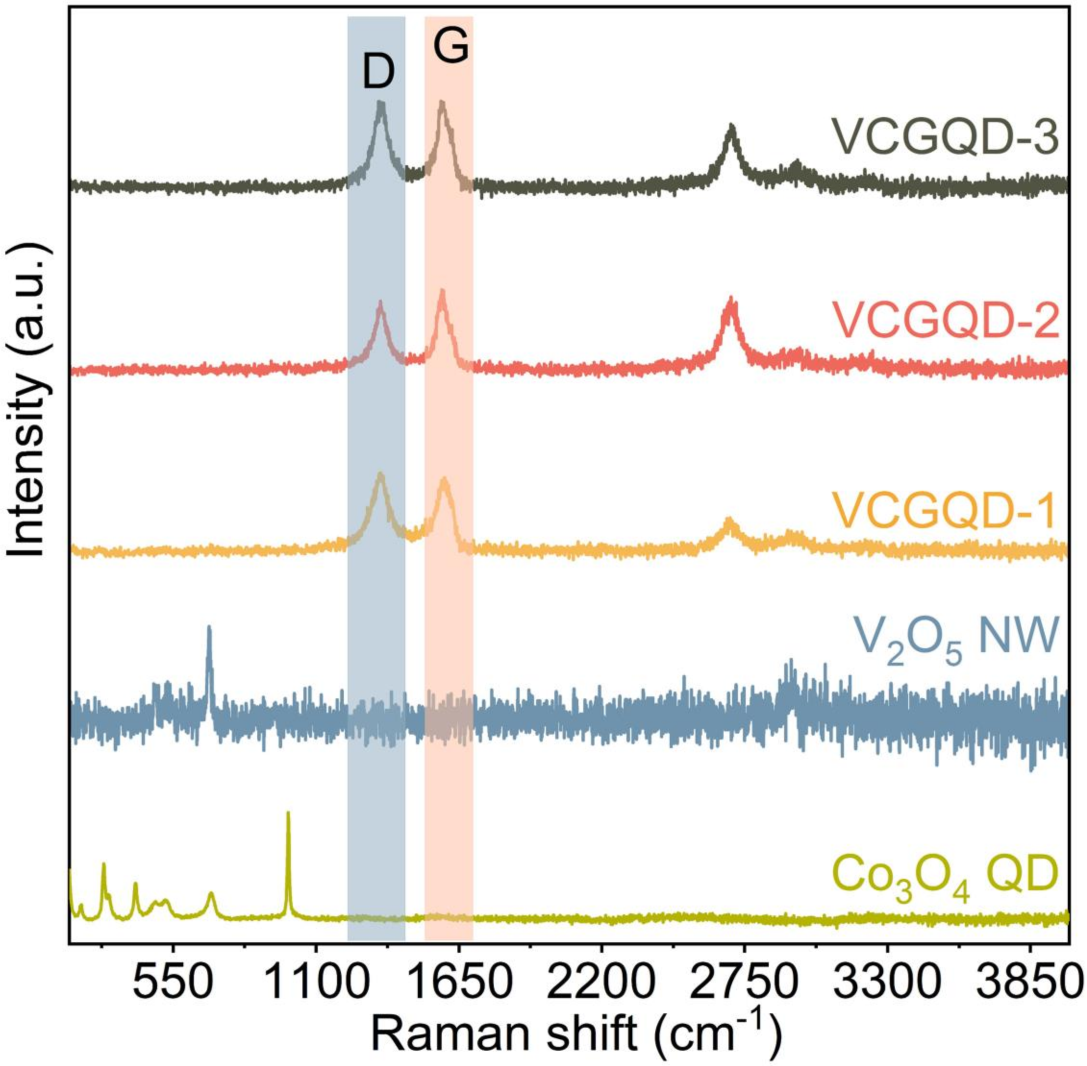

Figure S8. Raman spectra after ink reduction with different doping ratios of Co_3O_4 QDs.

11. After reduction: FT-IR

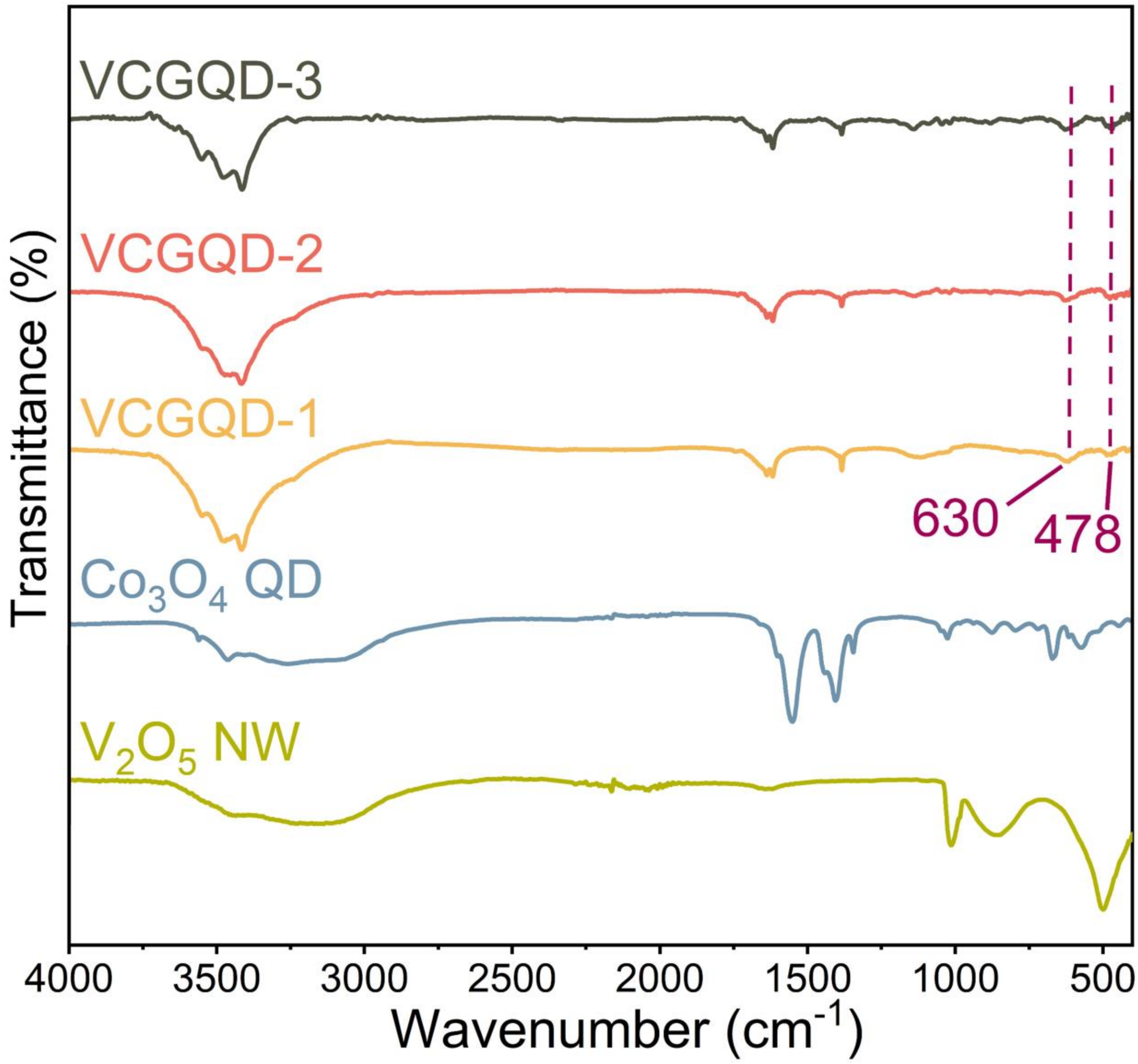

Figure S9. FT-IR spectra after ink reduction with different doping ratios of Co_3O_4 QDs..

12. Before reduction: XPS spectra of the

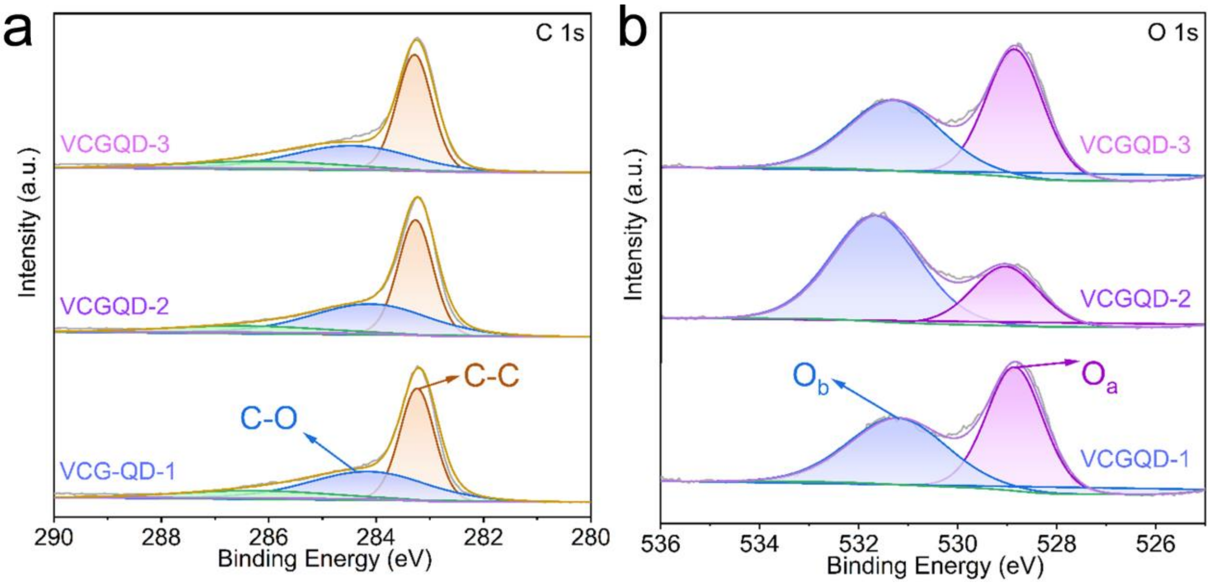

Figure S10. XPS spectra of the M2. a) Survey, and high resolution b) Ni 2p, c) O 1s, d) N 1s, e) C 1s and f) S 2p XPS spectra.

13. After reduction: XPS of all mixed gel

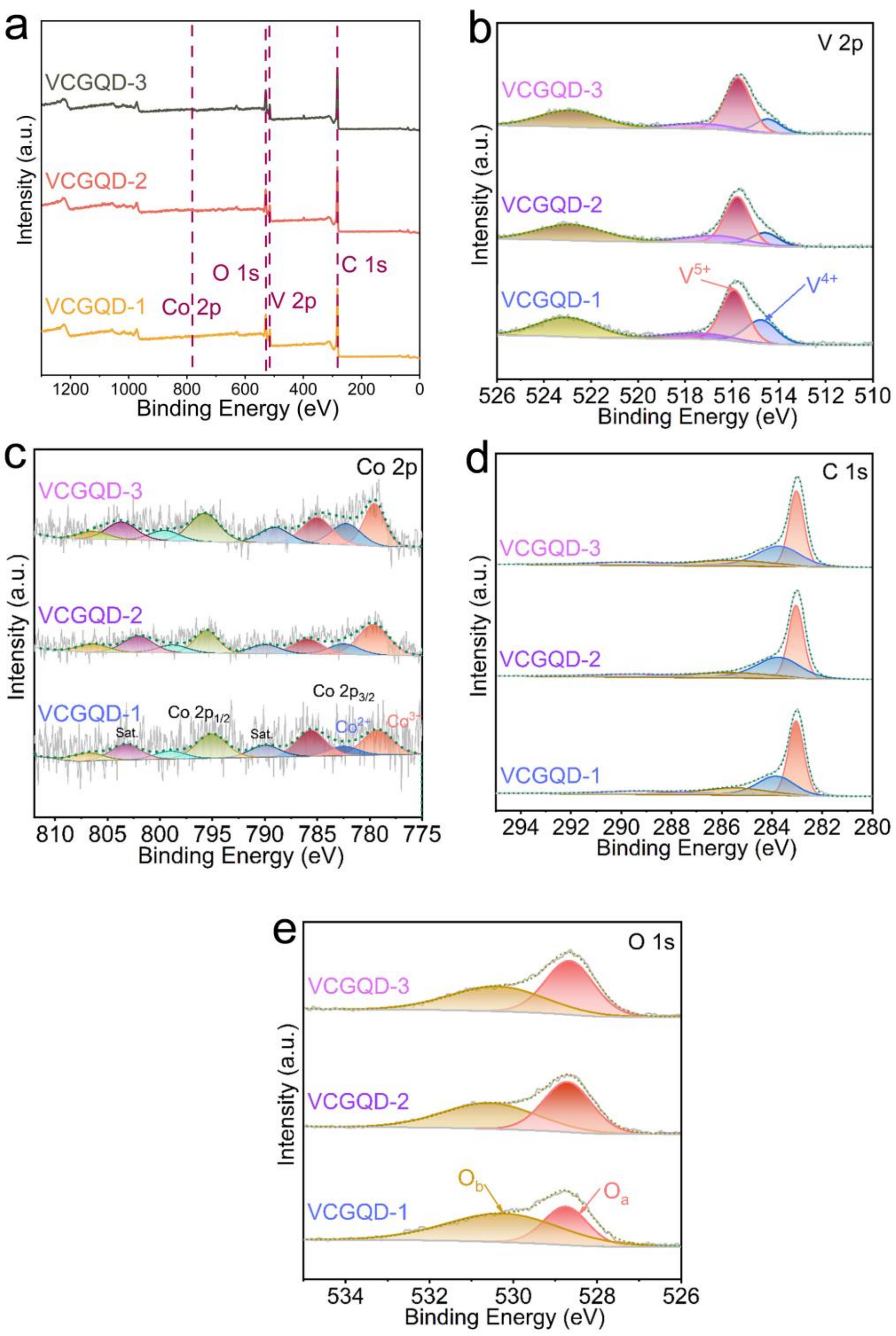

Figure S11. After reduction XPS spectra of all mixed gel. a) Survey; and high resolution b) V 2p; c) Co 2p; d) C 1s; e) O 1s.

14. Polarization microscope spectrogram under bright field conditions

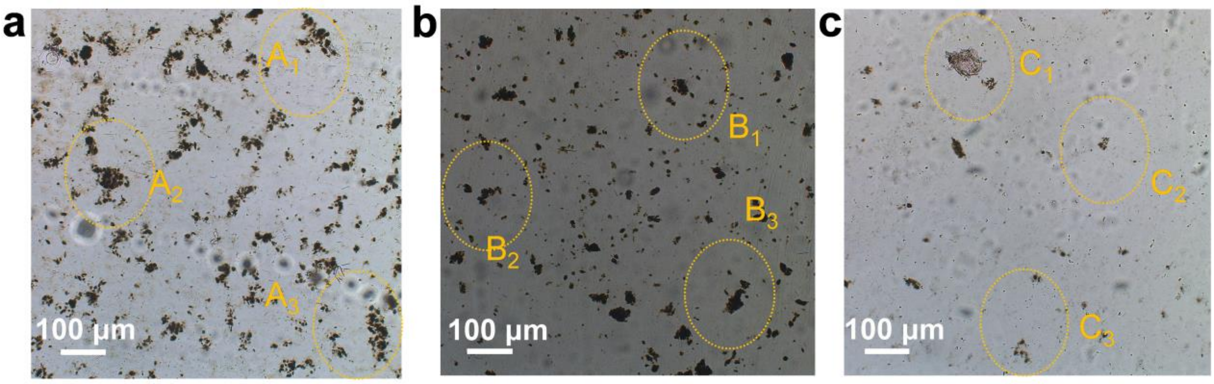

Figure S12. a) Polarization microscope spectrogram of VCGQD-1 mixed gel in bright field; b) Polarization microscope spectrogram of VCGQD-2 mixed gel in bright field; c) Polarization microscope spectrogram of VCGQD-3 mixed gel in bright field.

15. Polarization microscope spectrogram of VCG and VCGCDs

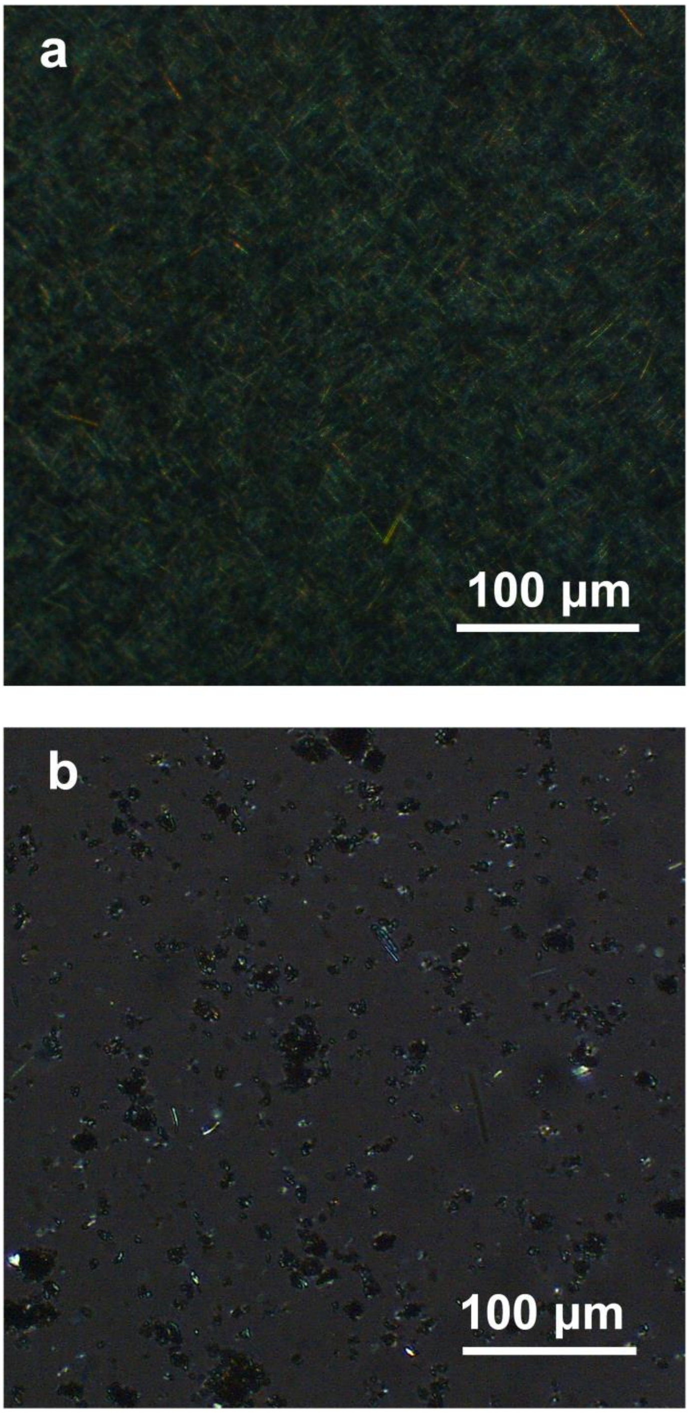

Figure S13. a) POM microscopic images of VCG mixed ink; b) VCGCDs mixed ink.

16. SEM image of VCG electrode cross-section

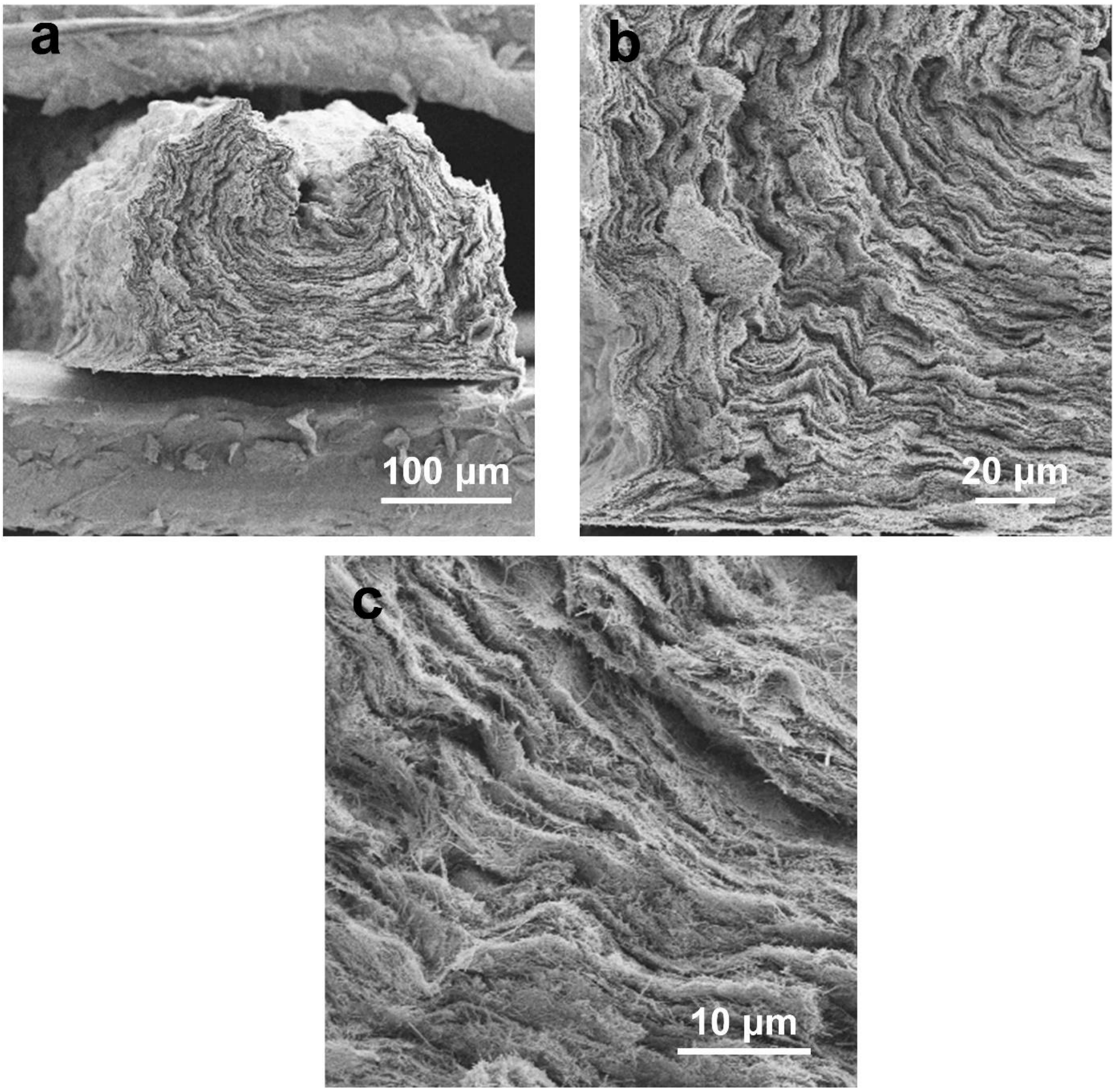

Figure S14. a, b and c) SEM image of VCG electrode cross-section.

17. Before reduction: XRD of VCGCDs

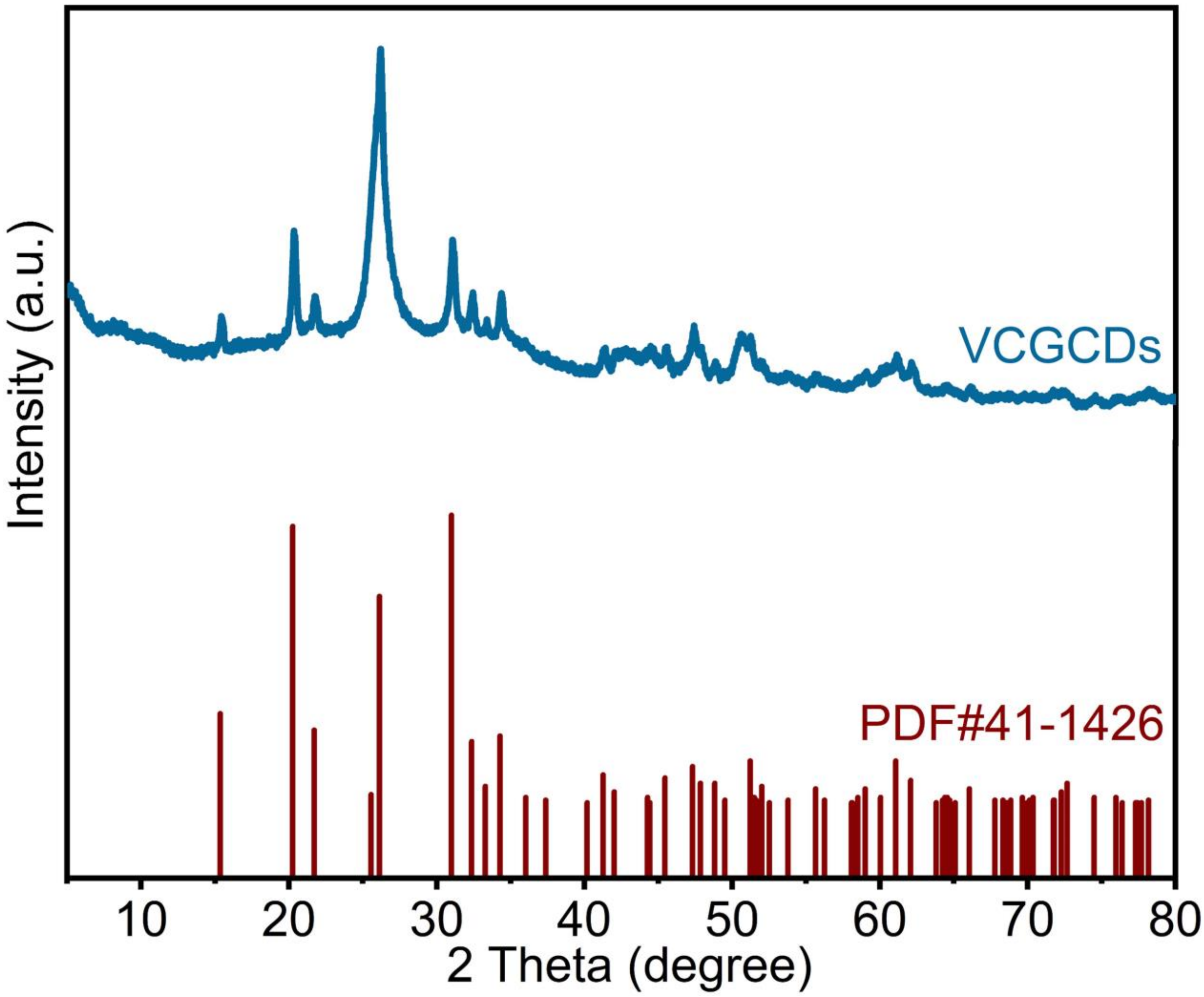

Figure S15. XRD of VCGCDs before reduction.

18. After reduction: XRD of VCGCDs

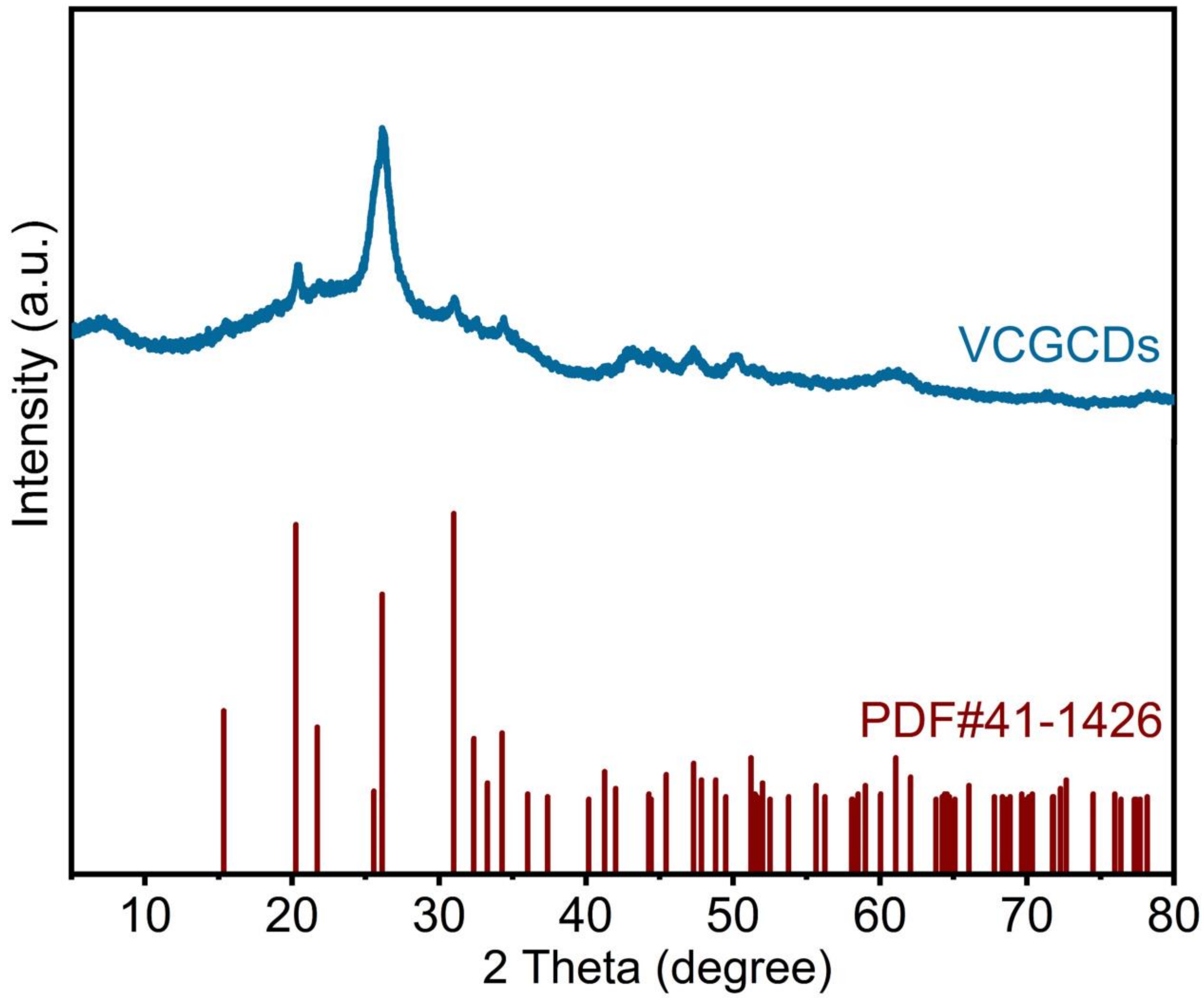

Figure S16. XRD of VCGCDs after reduction.

19. Rheological mechanics test of VCGCDs hydrogel

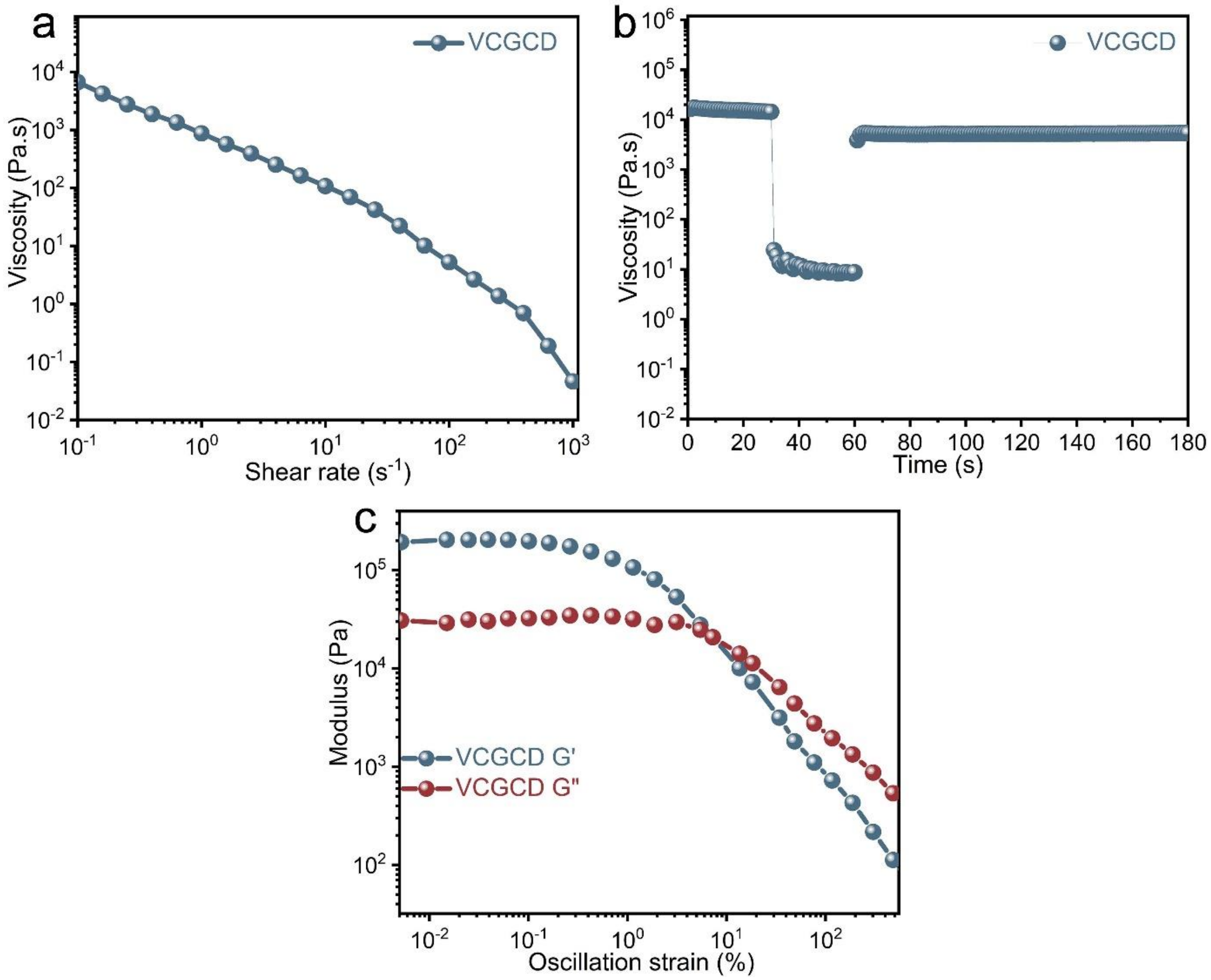

Figure S17. a) Apparent viscosity of the VCGCDs hydrogels as a function of shear rate; b) PHS experiment; c) The G′ and G″ of VCGCDs hydrogels.

20. TEM of VCGCDs mixed hydrogel

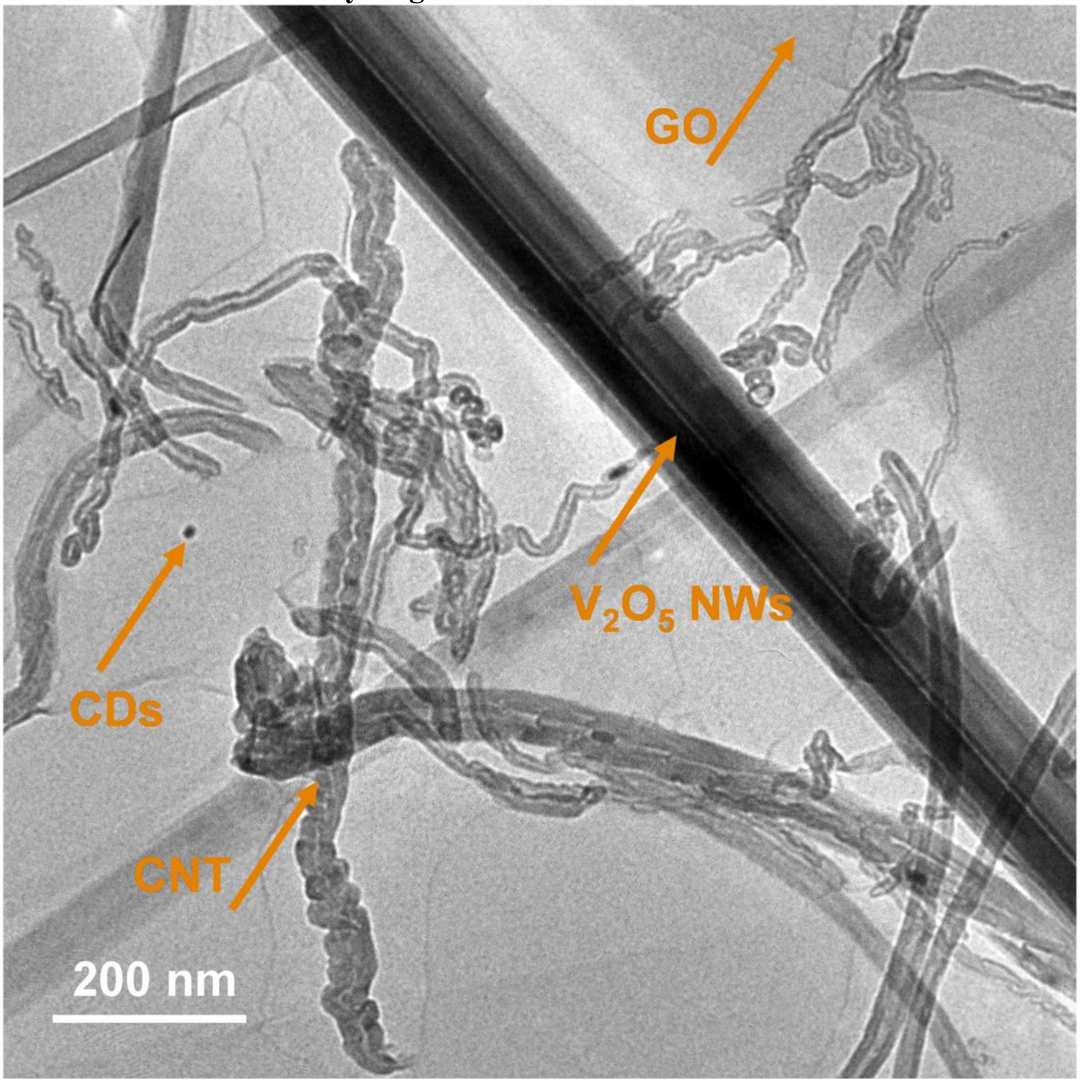

Figure S18. TEM of VCGCDs mixed hydrogel.

21. SEM of VCGCDs mixed hydrogel

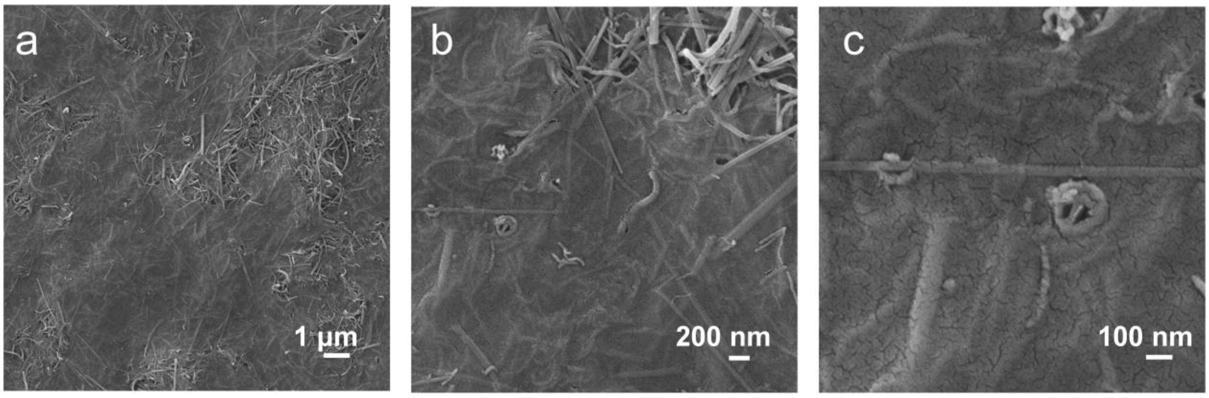

Figure S19. a, b and c) SEM images of VCGCDs mixed hydrogels at different magnification.

22. SEM image of VCGCDs electrode cross-section

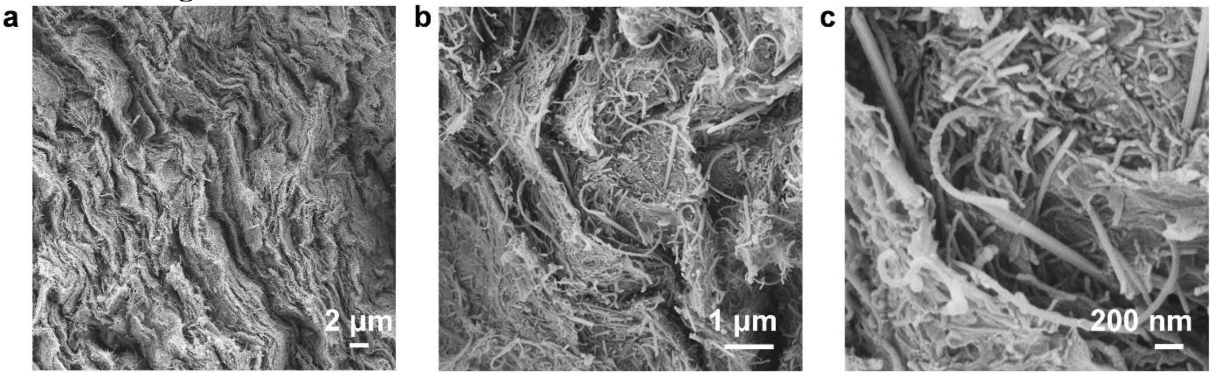

Figure S20. a, b and c) SEM images of VCGCDs electrode cross-section at different magnification.

23. The analysis of ion-diffusion and capacitive contributions of the VCGQD-1

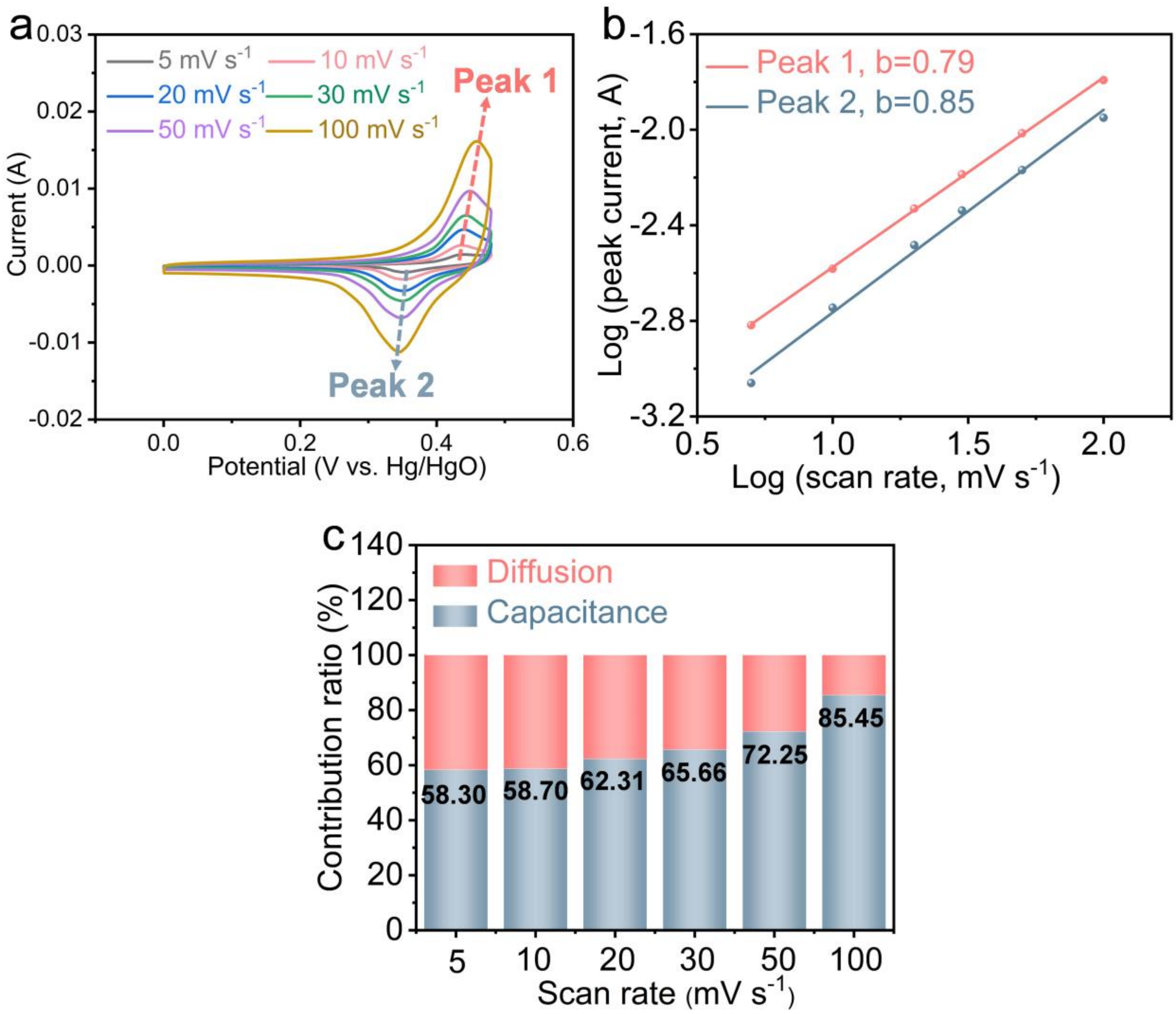

Figure S21. a) CV curves of the VCGQD-1 at various scan rates of 5-100 mV s^{-1} in a three-electrode cell; b) Log*(i)* versus log*(v)* plots of the VCGQD-1 at specific peak currents; c) Bar chart showing the percent of pseudocapacitive contribution of the VCGQD-1 at different scan rates.

24. Pseudocapacitive contribution shadow diagram of VCGQD-1 in CV curves

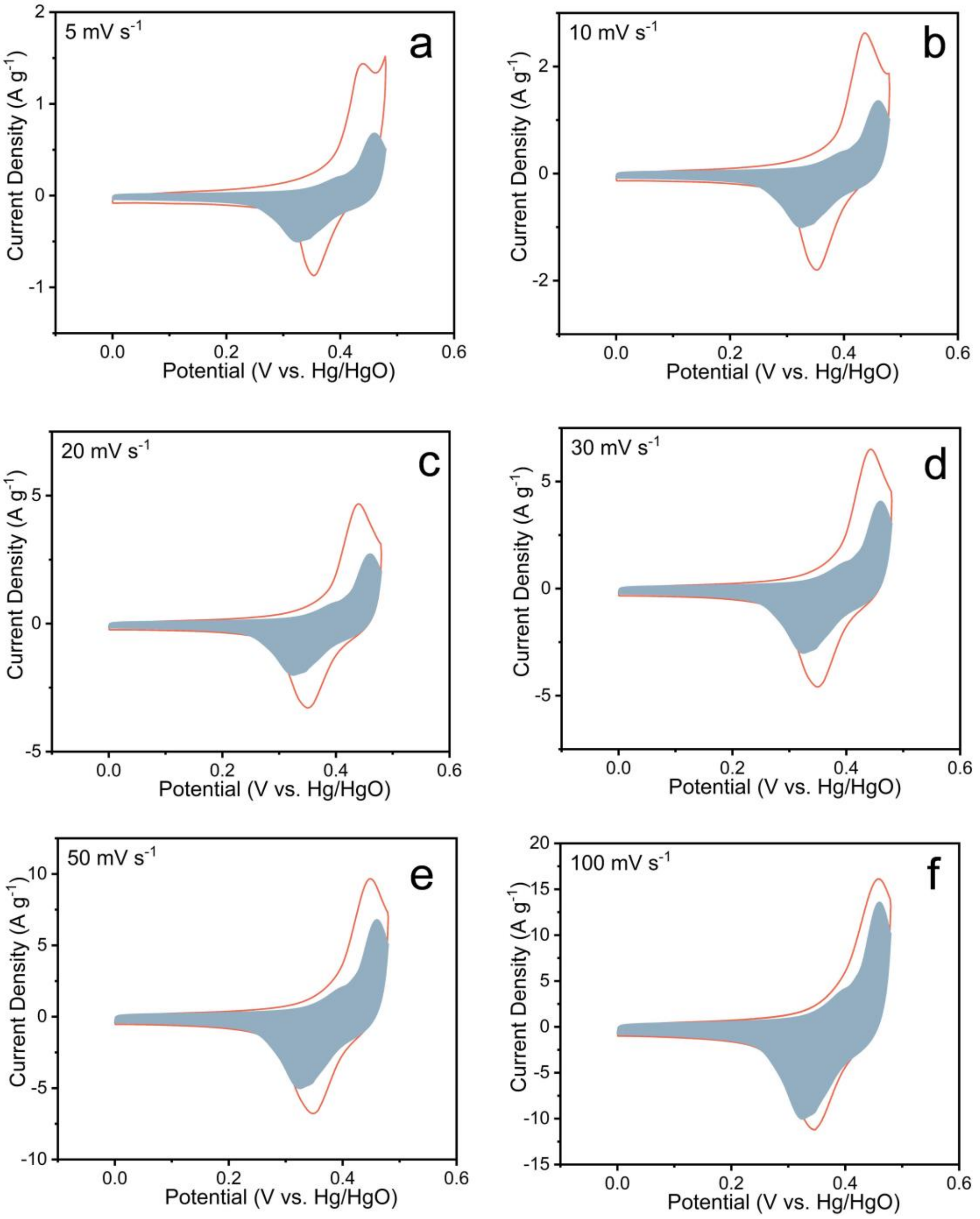

Figure S22. CV curve with the pseudocapacitive fraction shown by the shaded area of M8 at various scan rates in a three-electrode cell. a) 5 mV s^{-1}. b) 10 mV s^{-1}. c) 20 mV s^{-1}. d) 30 mV s^{-1}. e) 50 mV s^{-1}. f) 100 mV s^{-1}.

25. The analysis of ion-diffusion and capacitive contributions of the VCGQD-2

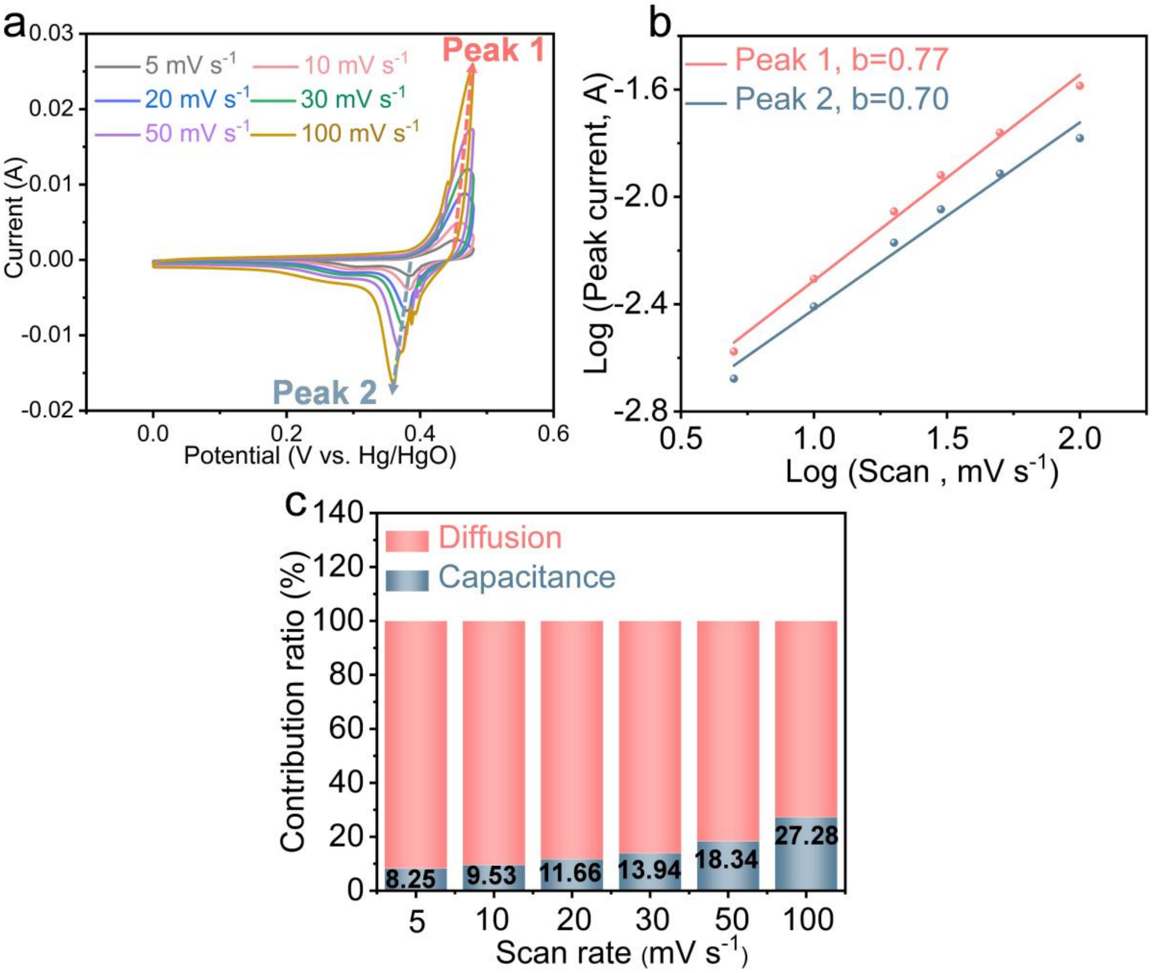

Figure S23. a) CV curves of the VCGQD-2 at various scan rates of 5-100 mV s^{-1} in a three-electrode cell. b) Log*(i)* versus log*(v)* plots of the VCGQD-2 at specific peak currents. c) Bar chart showing the percent of pseudocapacitive contribution of the VCGQD-2 at different scan rates.

26. Pseudocapacitive contribution shadow diagram of VCGQD-2 in CV curves

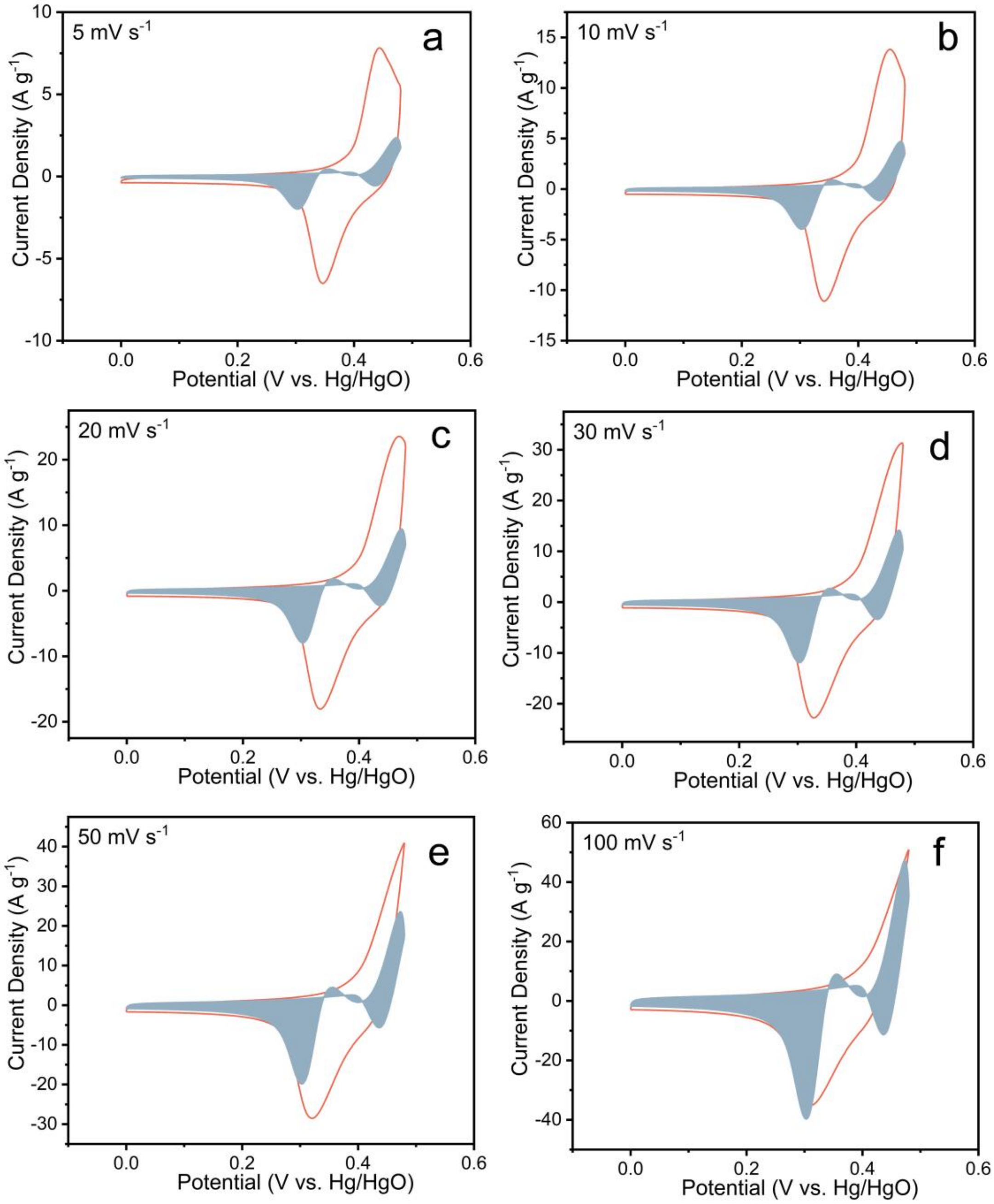

Figure S24. CV curve with the pseudocapacitive fraction shown by the shaded area of VCGQD-2 at various scan rates in a three-electrode cell. a) 5 mV s^{-1}. b) 10 mV s^{-1}. c) 20 mV s^{-1}. d) 30 mV s^{-1}. e) 50 mV s^{-1}. f) 100 mV s^{-1}.

27. The analysis of ion-diffusion and capacitive contributions of the VCGQD-3

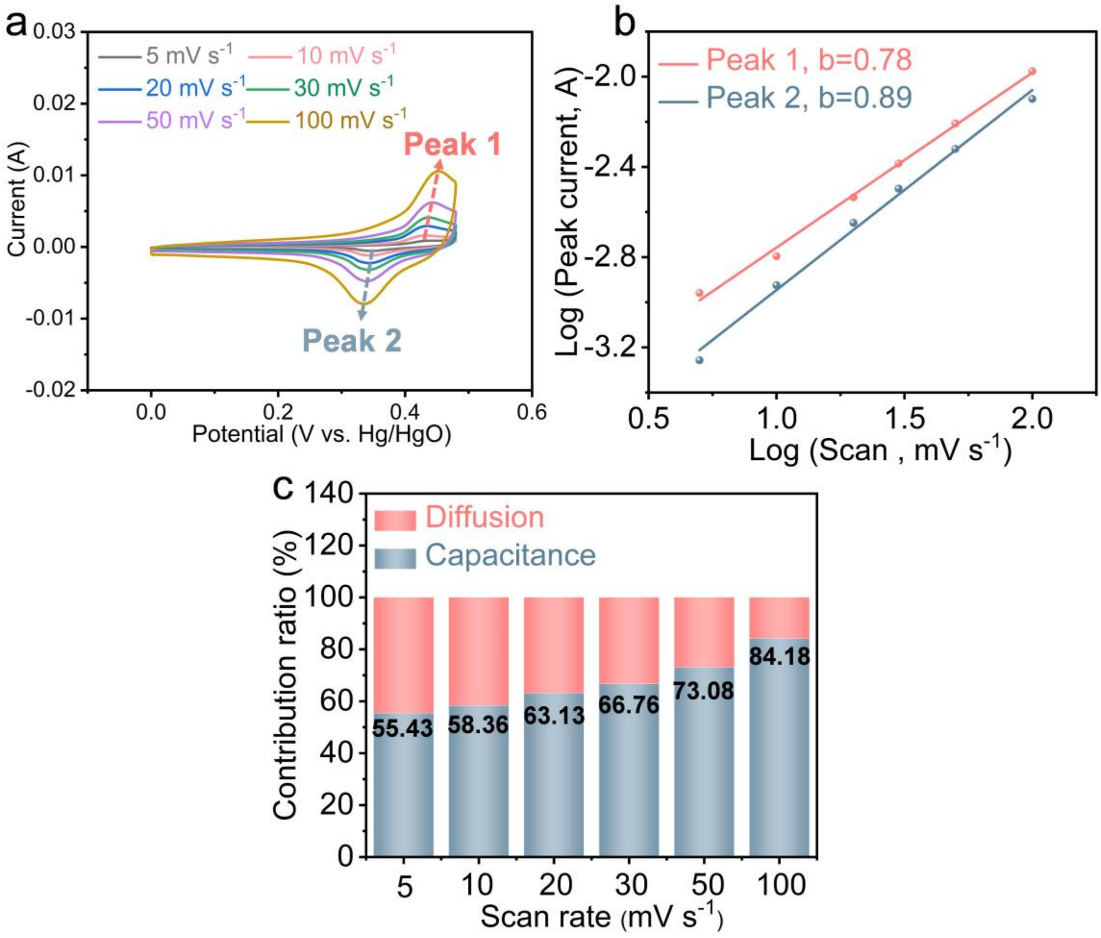

Figure S25. a) CV curves of the VCGQD-3 at various scan rates of 5-100 mV s^{-1} in a three-electrode cell. b) Log*(i)* versus log*(v)* plots of the VCGQD-3 at specific peak currents. c) Bar chart showing the percent of pseudocapacitive contribution of the VCGQD-3 at different scan rates.

28. Pseudocapacitive contribution shadow diagram of VCGQD-3 in CV curves

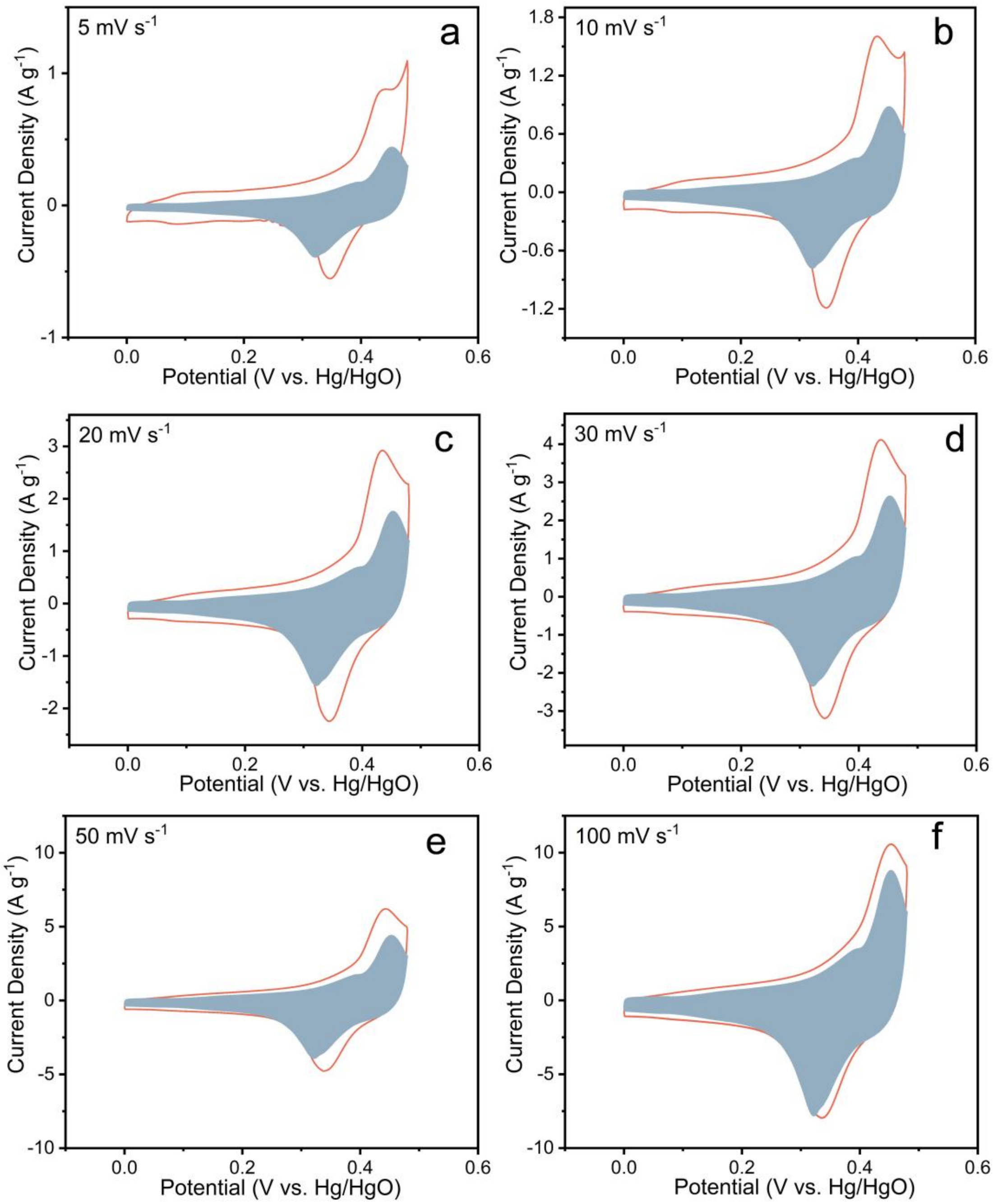

Figure S26. CV curve with the pseudocapacitive fraction shown by the shaded area of VCGQD-3 at various scan rates in a three-electrode cell. a) 5 mV s^{-1}. b) 10 mV s^{-1}. c) 20 mV s^{-1}. d) 30 mV s^{-1}. e) 50 mV s^{-1}. f) 100 mV s^{-1}.

29. Three-electrode systems: the GCD curves of VCGQD-1 and VCGQD-3 at different current densities

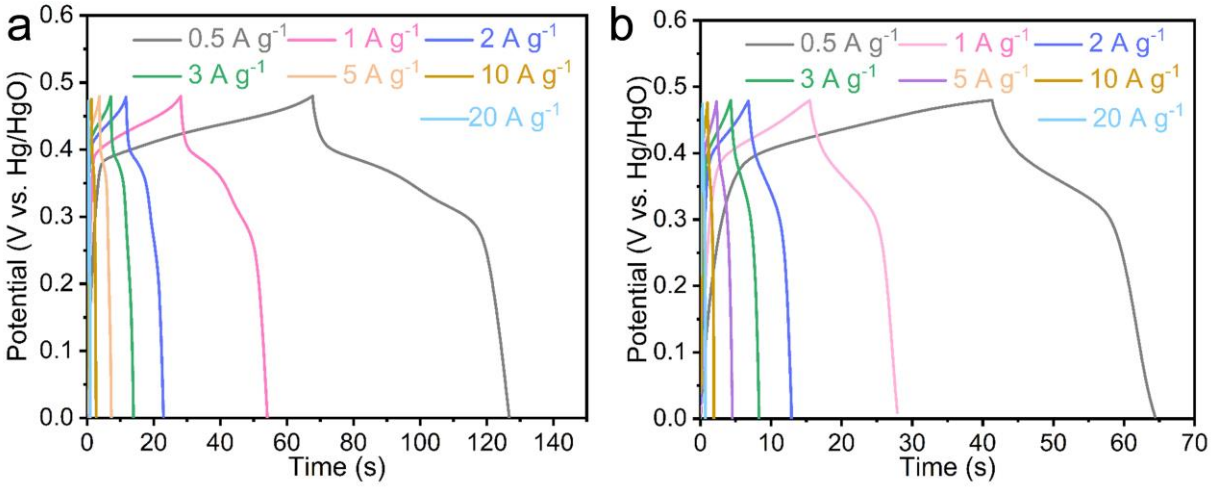

Figure S27. a) GCD curves of VCGQD-1 and b) VCGQD-3 at different current densities in a three-electrode cell.

30. CV and GCD curves of the VCGQD//MXene at sample potentials and scan rate

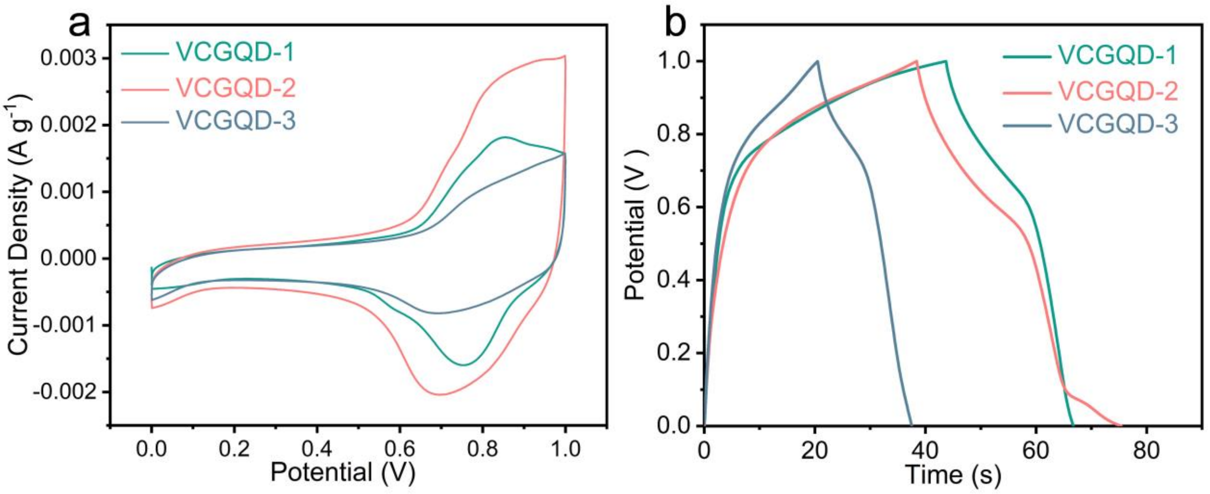

Figure S28. a) CV and b) GCD curves of the VCGQD//MXene at sample potentials and scan rate.

31. The analysis of ion-diffusion and capacitive contributions of the VCGQD-1//MXene

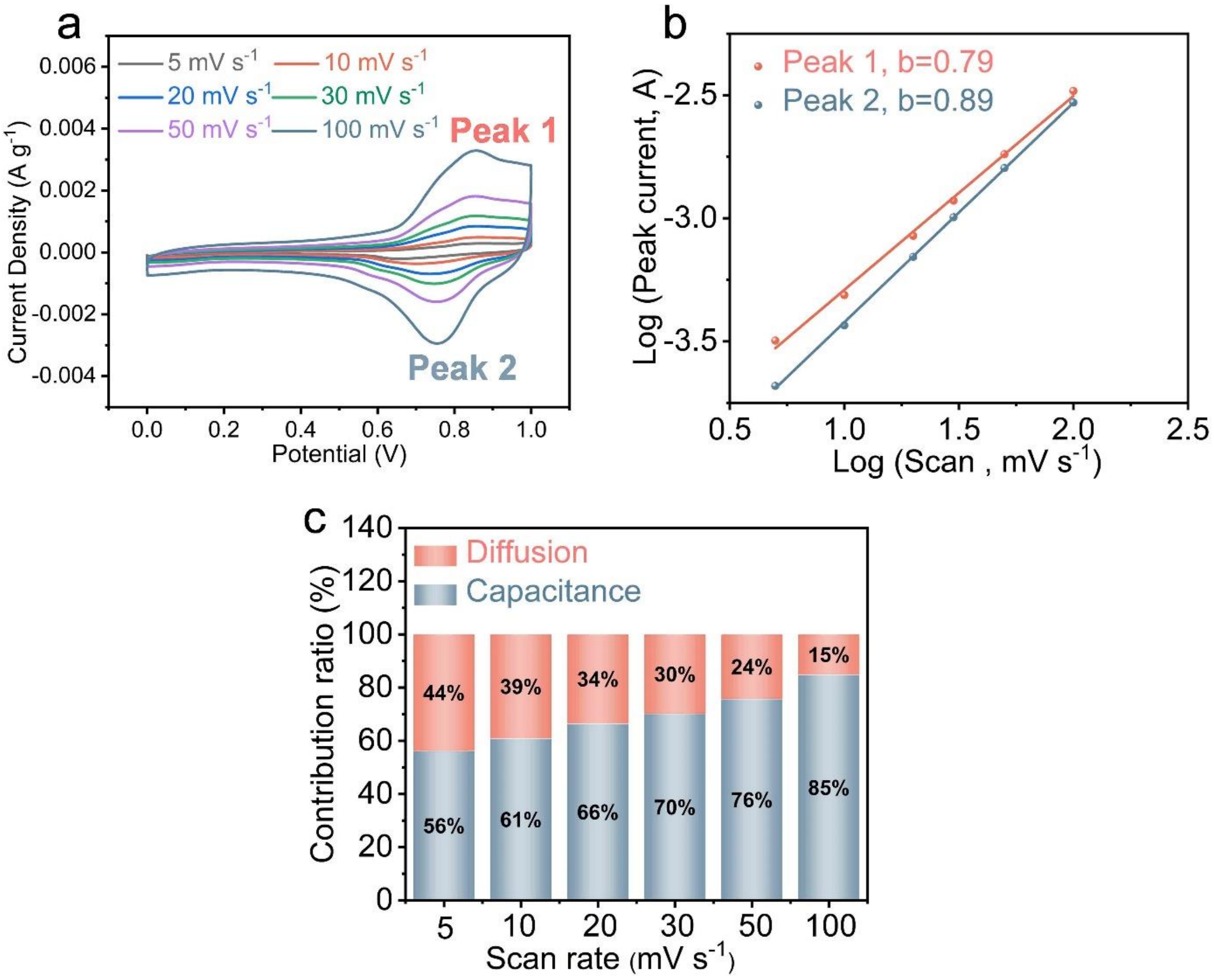

Figure S29. a) CV curves of the VCGQD-1//MXene at various scan rates of 5-100 mV s^{-1} in a three-electrode cell. b) Log*(i)* versus log*(v)* plots of the VCGQD-1//MXene at specific peak currents. c) Bar chart showing the percent of pseudocapacitive contribution of the VCGQD-1//MXene at different scan rates.

32. Pseudocapacitive contribution shadow diagram of VCGQD-1//MXene in CV curves

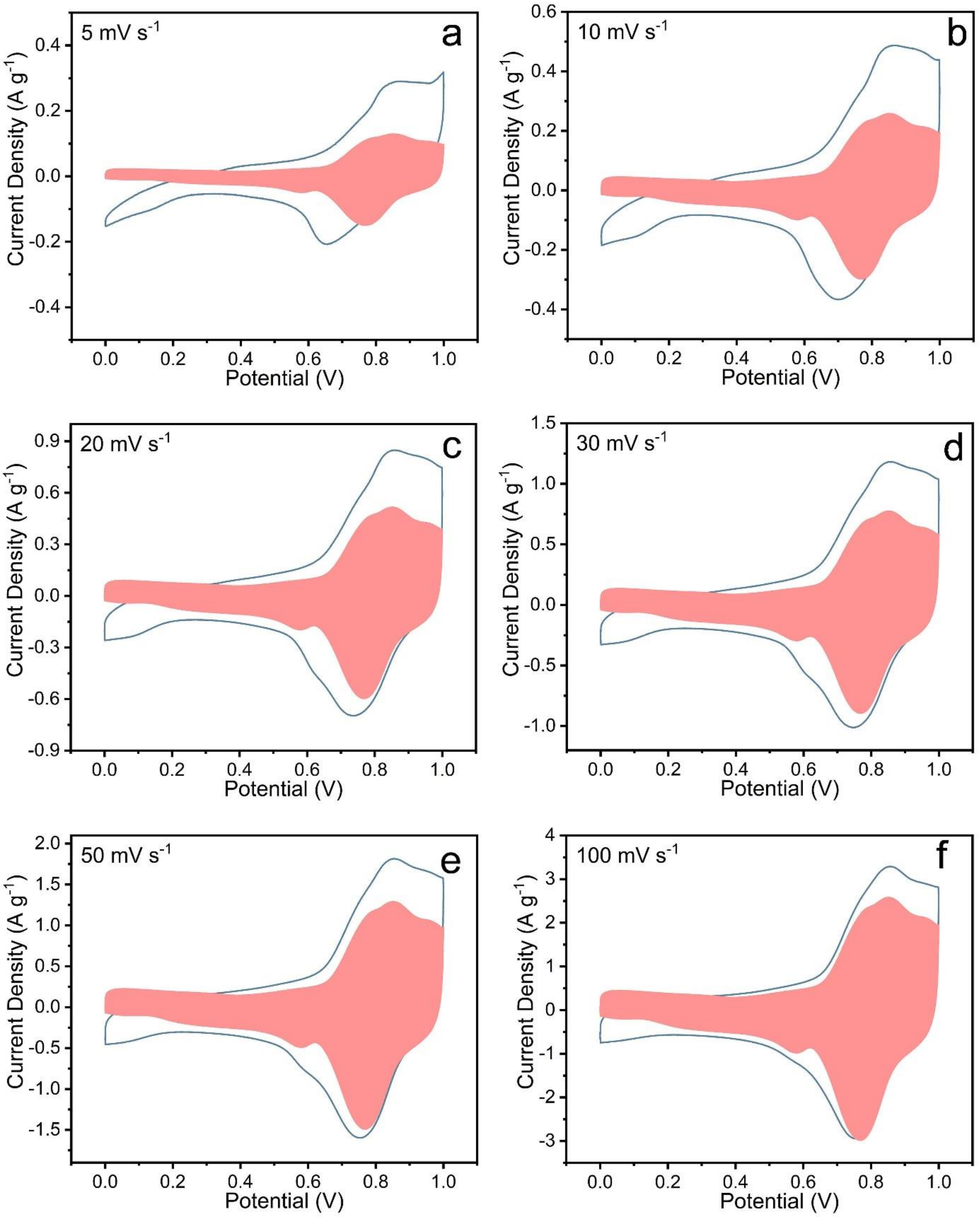

Figure S30. CV curve with the pseudocapacitive fraction shown by the shaded area of VCGQD-1//MXene at various scan rates. a) 5 mV s^{-1}. b) 10 mV s^{-1}. c) 20 mV s^{-1}. d) 30 mV s^{-1}. e) 50 mV s^{-1}. f) 100 mV s^{-1}.

33. The analysis of ion-diffusion and capacitive contributions of the VCGQD-2//MXene

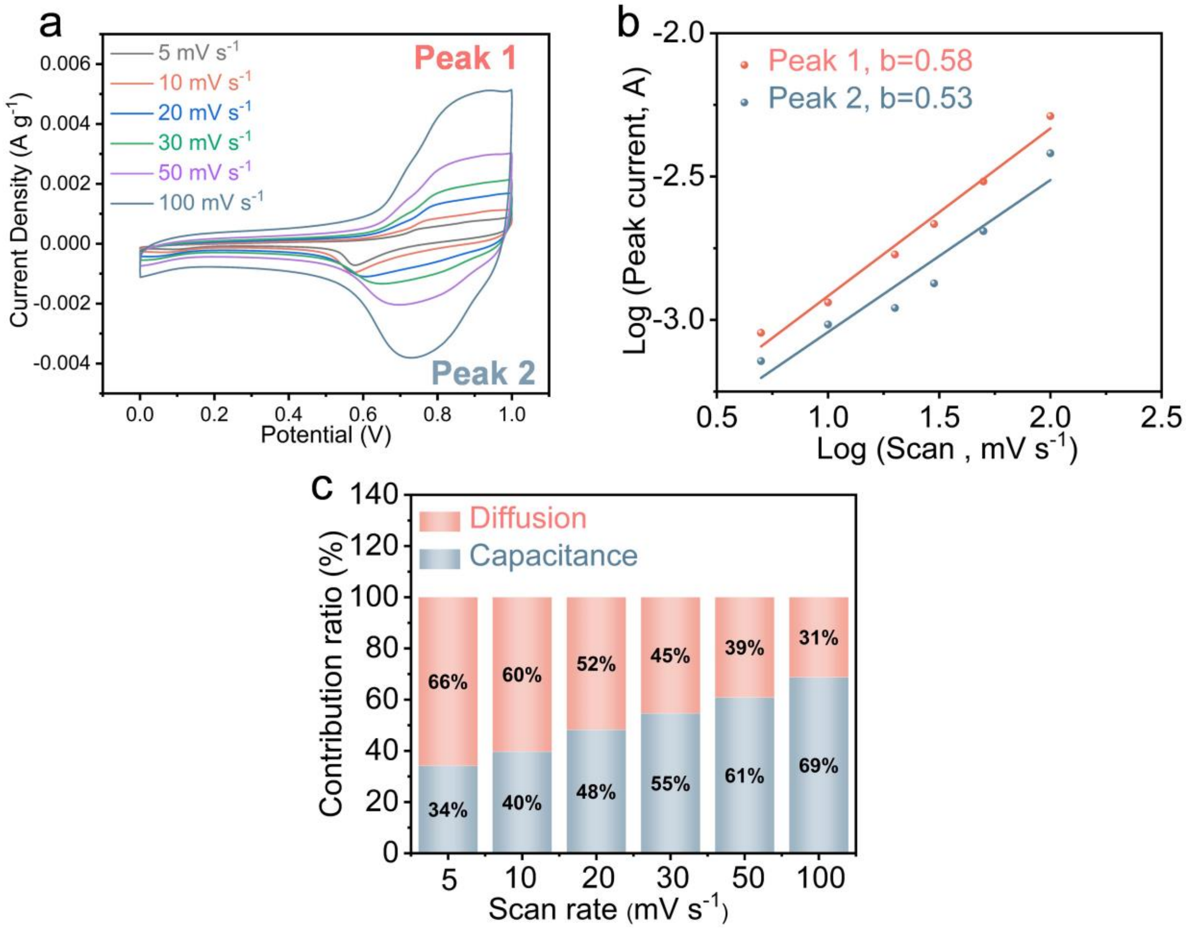

Figure S31. a) CV curves of the VCGQD-2//MXene at various scan rates of 5-100 mV s^{-1} in a two-electrode cell. b) Log*(i)* versus log*(v)* plots of the VCGQD-2//MXene at specific peak currents. c) Bar chart showing the percent of pseudocapacitive contribution of the VCGQD-2//MXene at different scan rates.

34. Pseudocapacitive contribution shadow diagram of VCGQD-2//MXene in CV curves

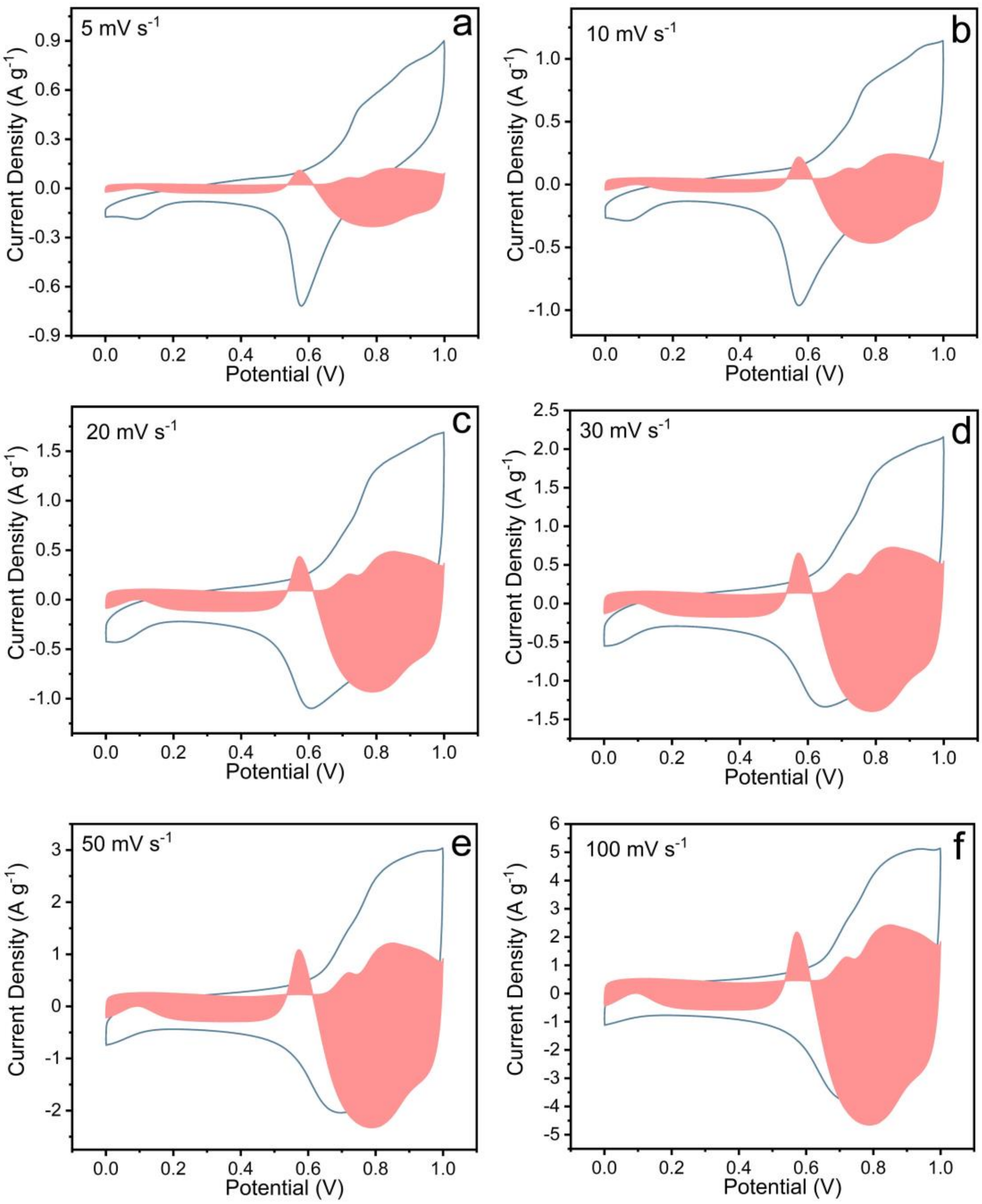

Figure S32. CV curve with the pseudocapacitive fraction shown by the shaded area of VCGQD-2//MXene at various scan rates. a) 5 mV s^{-1}. b) 10 mV s^{-1}. c) 20 mV s^{-1}. d) 30 mV s^{-1}. e) 50 mV s^{-1}. f) 100 mV s^{-1}.

35. The analysis of ion-diffusion and capacitive contributions of the VCGQD-3//MXene

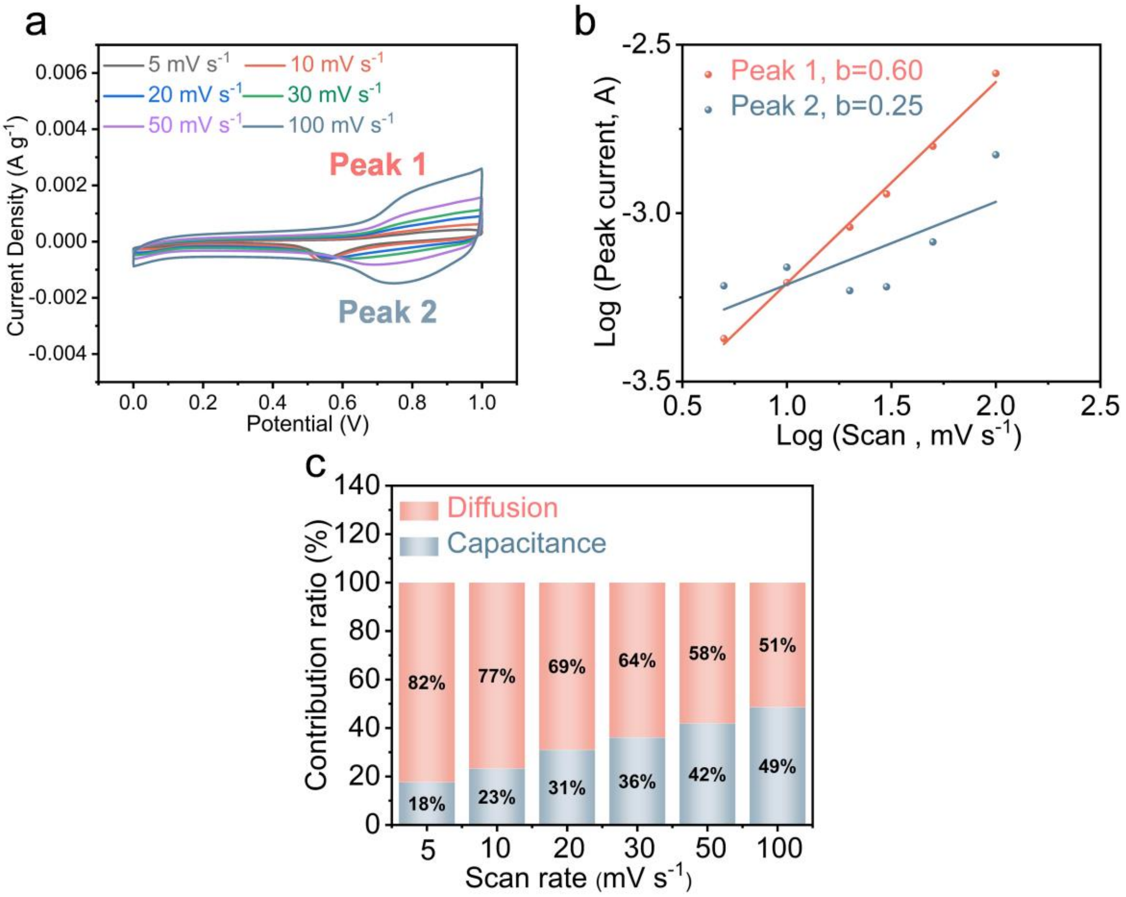

Figure S33. a) CV curves of the VCGQD-3//MXene at various scan rates of 5-100 mV s^{-1} in a three-electrode cell. b) Log*(i)* versus log*(v)* plots of the VCGQD-3//MXene at specific peak currents. c) Bar chart showing the percent of pseudocapacitive contribution of the VCGQD-3//MXene at different scan rates.

36. Pseudocapacitive contribution shadow diagram of VCGQD-3//MXene in CV curves

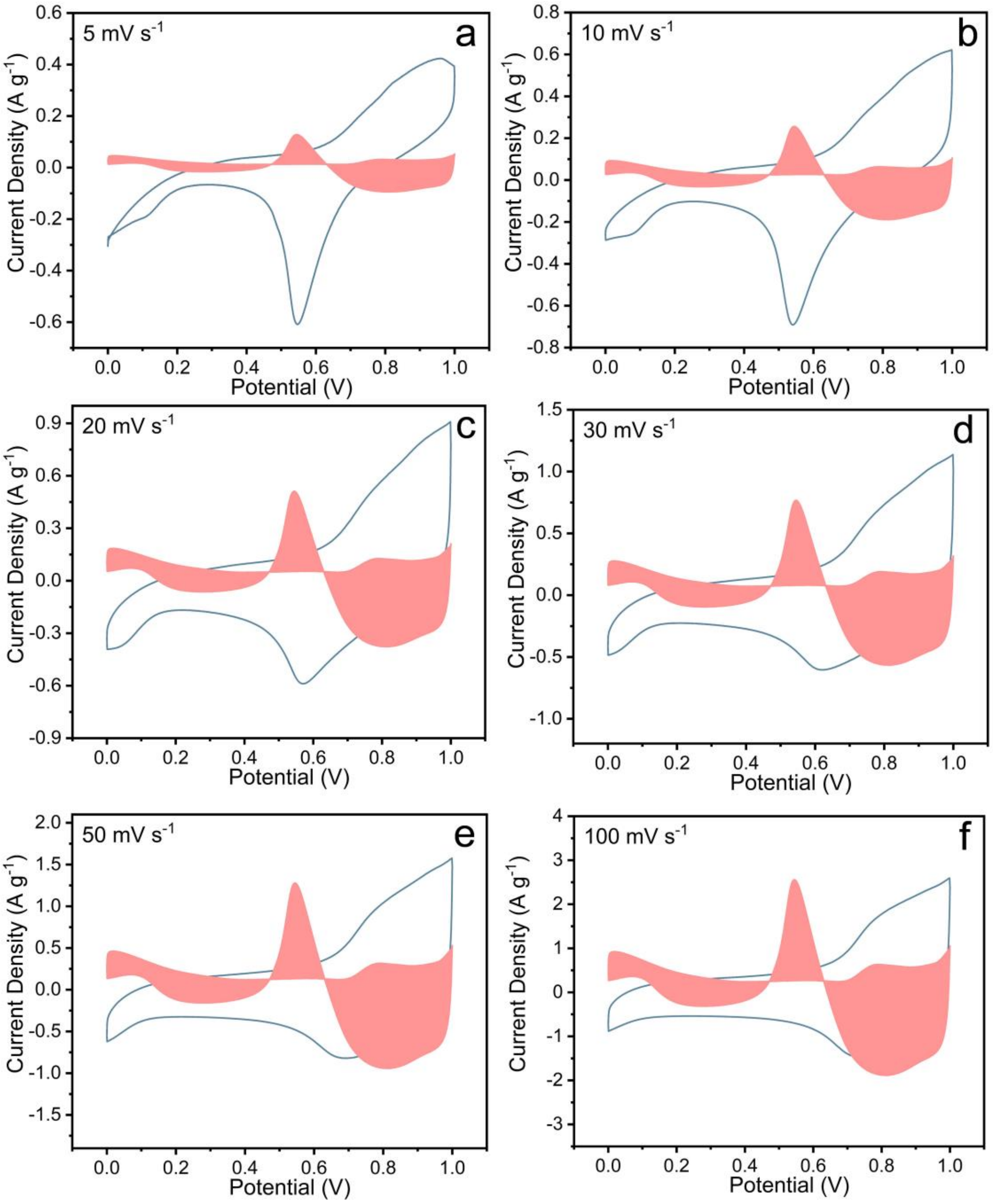

Figure S34. CV curve with the pseudocapacitive fraction shown by the shaded area of VCGQD-3//MXene at various scan rates. a) 5 mV s^{-1}. b) 10 mV s^{-1}. c) 20 mV s^{-1}. d) 30 mV s^{-1}. e) 50 mV s^{-1}. f) 100 mV s^{-1}.

37. Two-electrode: CV and GCD curves of the VCGQD//MXene at different potentials

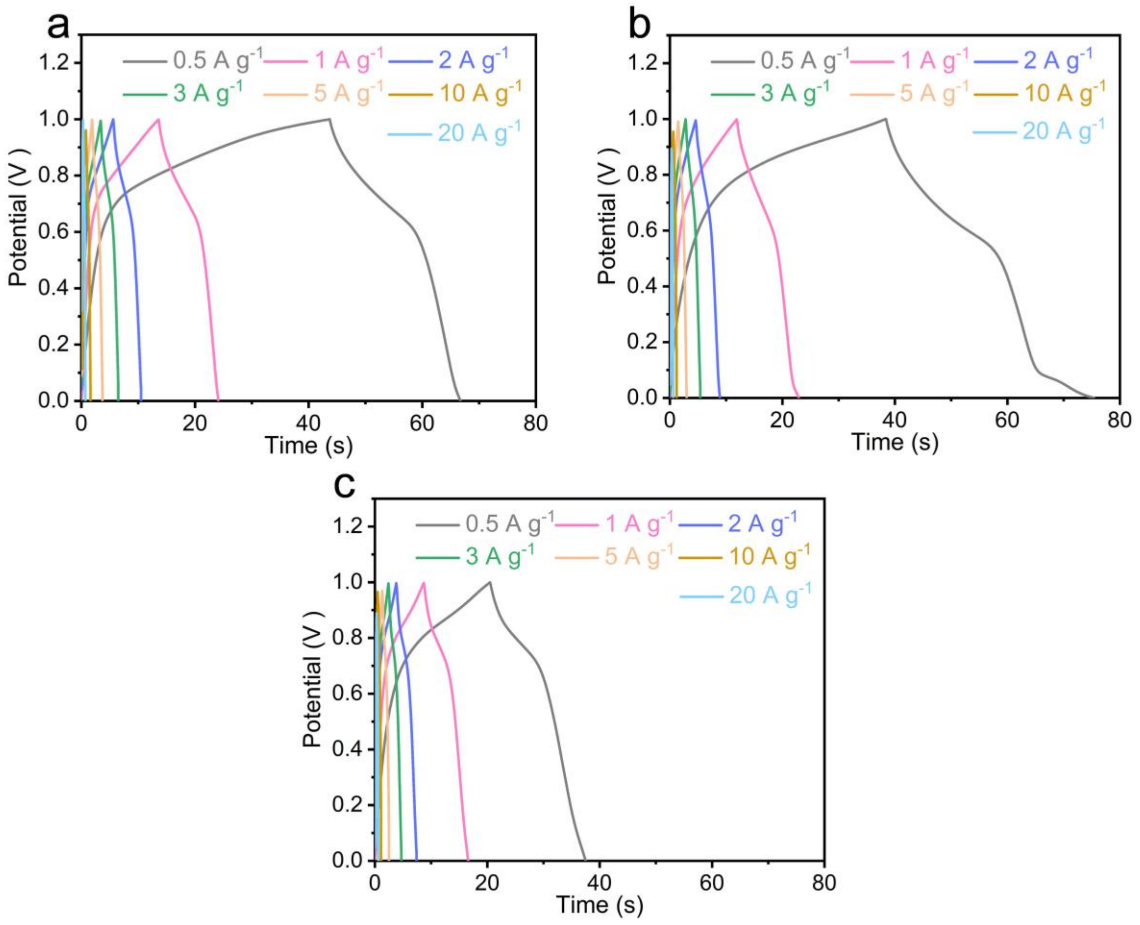

Figure S35. The GCD curves at different current densities in a two-electrode cell. a) VCGQD-1; b) VCGQD-2 and c) VCGQD-3.

38. CV curves of the VCGQD-1//Mxene and VCGQD-3//Mxene MSCs at scan rates

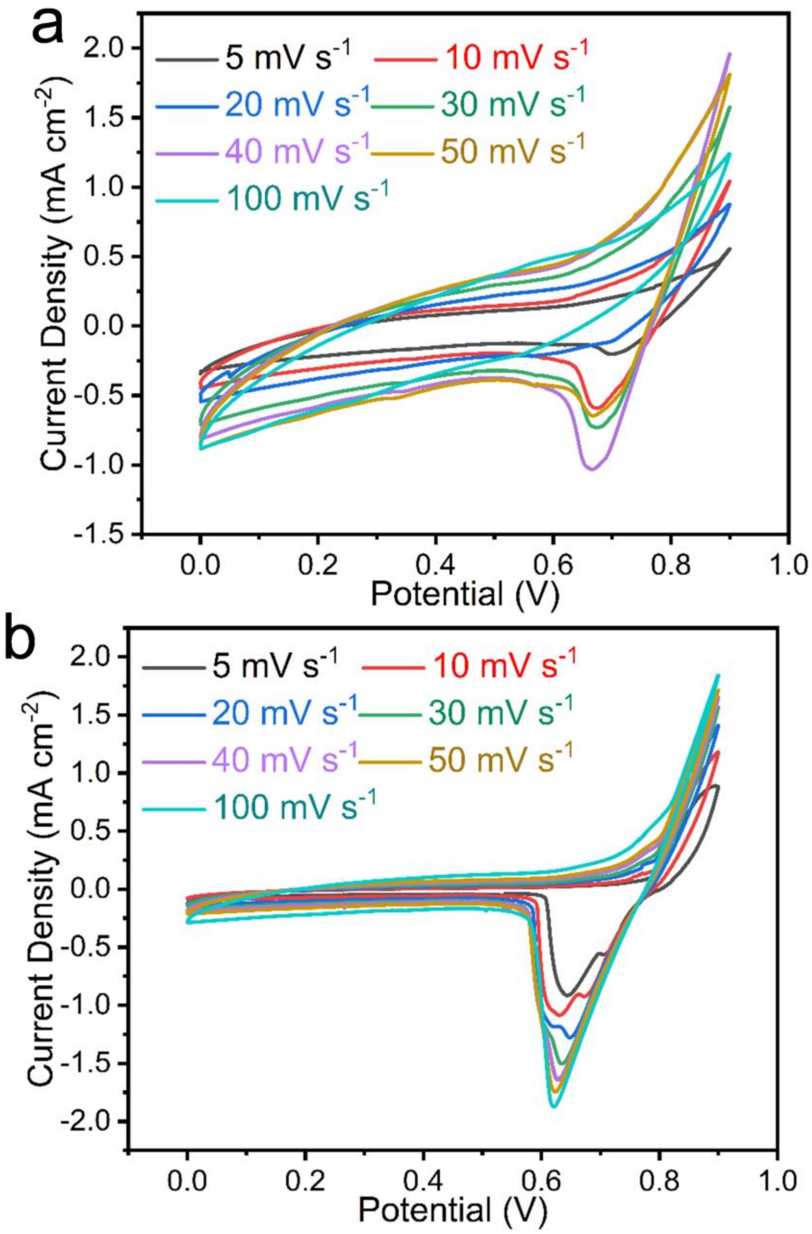

Figure S36. The GCD curves at different current densities. a) VCGQD-1//MXene MSC; b) VCGQD-3//MXene MSC.

39. GCD curves of the VCGQD-1//Mxene and VCGQD-3//Mxene MSCs at different current density

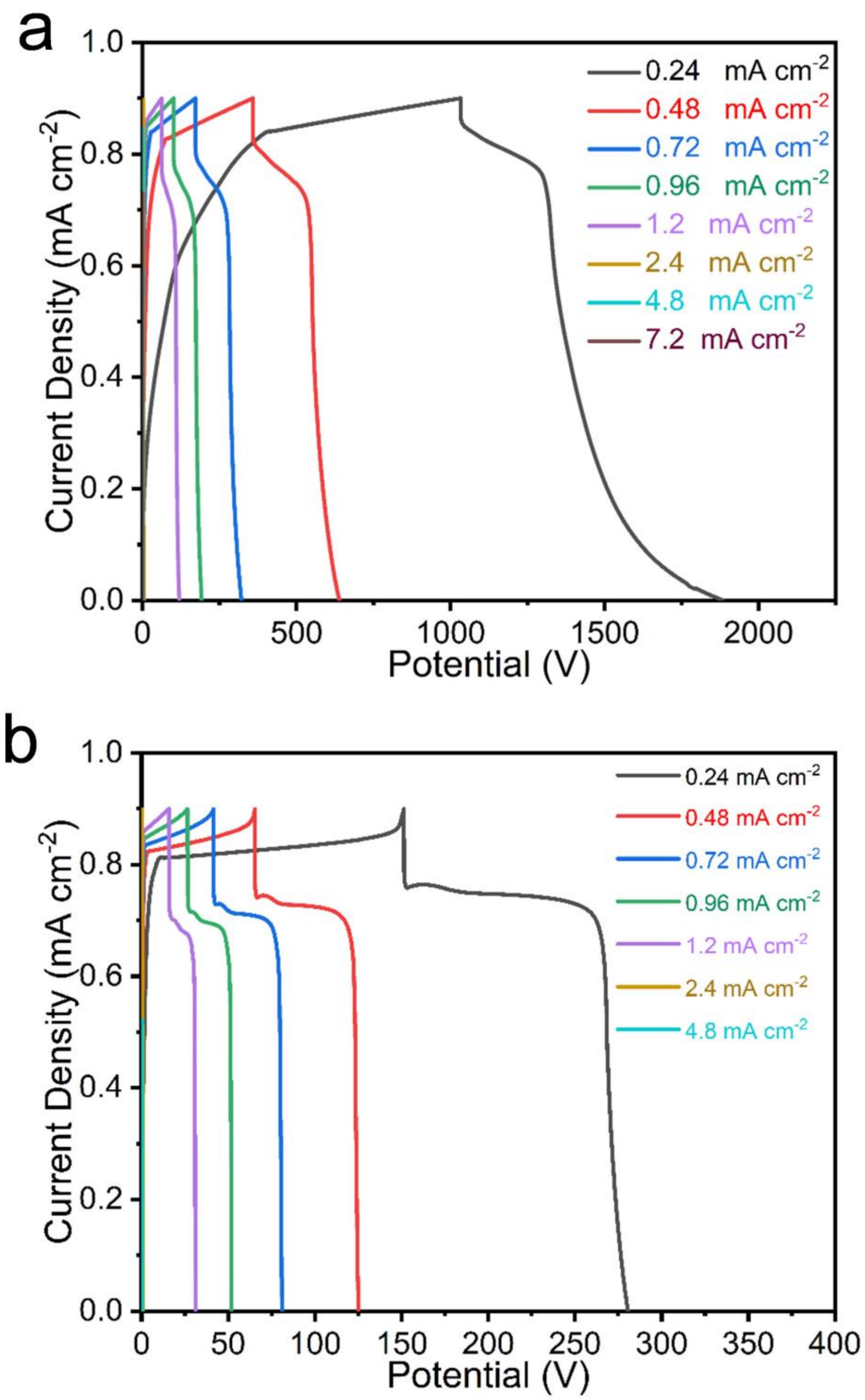

Figure S37. The GCD curves at different current densities. a) VCGQD-1//Mxene and b) VCGQD-3//Mxene MSCs.

40. Electrochemical characterization of the VCGQD-2//MXene MSC

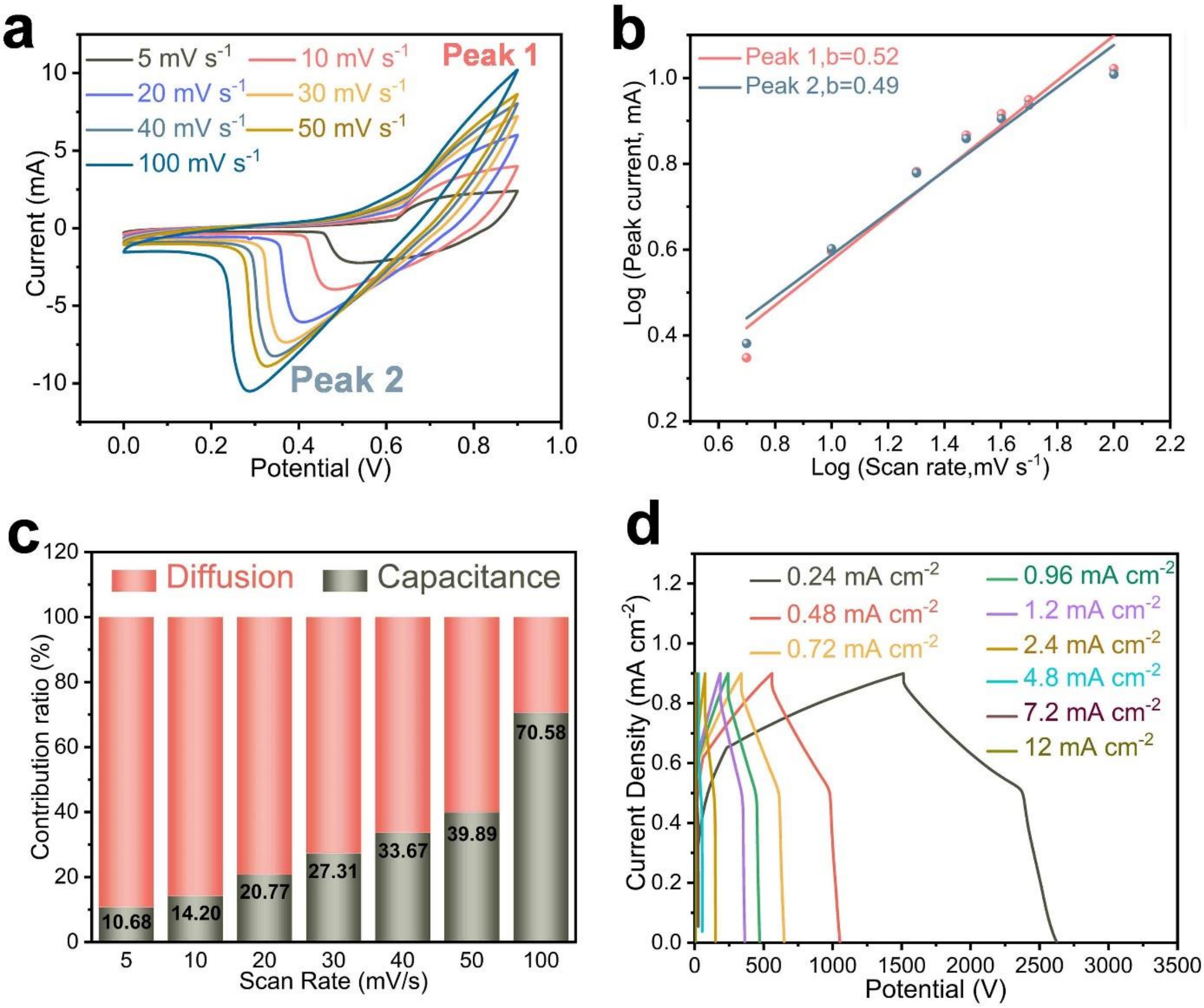

Figure S38. a) CV curves of the VCGQD-3//MXene at various scan rates of 5-100 mV s^{-1} in a three-electrode cell; b) Log(i) versus log(v) plots of the VCGQD-3//MXene at specific peak currents; c) Bar chart showing the percent of pseudocapacitive contribution of the VCGQD-3//MXene at different scan rates; d) The GCD curves of VCGQD-2//Mxene at different current densities.

41. CV and GCD curves of the VCGCDs//MXene

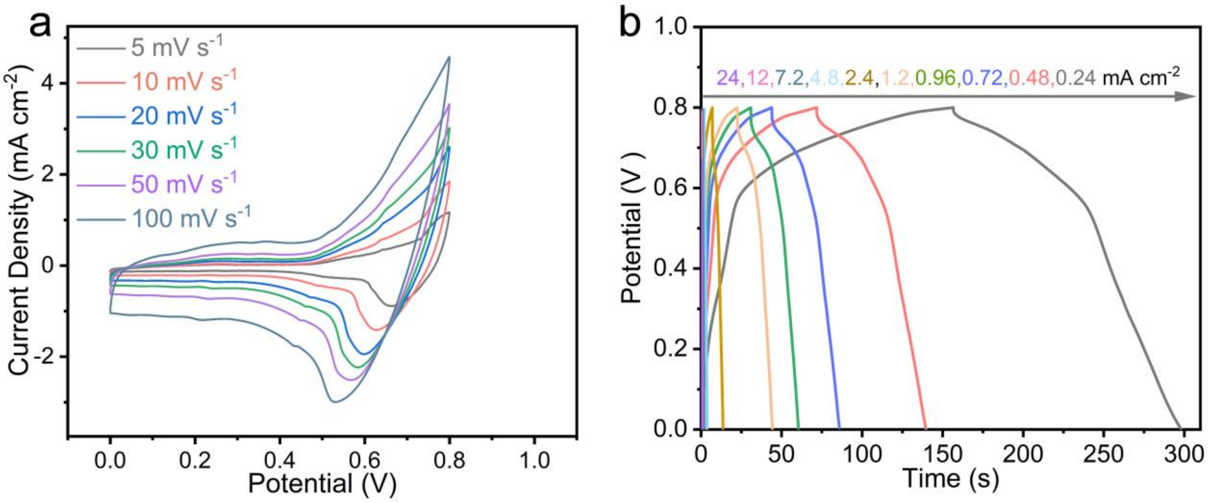

Figure S39. a) CV curves of the VCGCDs//MXene at different scan rates; b) The GCD curves of VCGCDs//MXene at different current density.

42. CV and GCD curves of the Co_3O_4 QDs

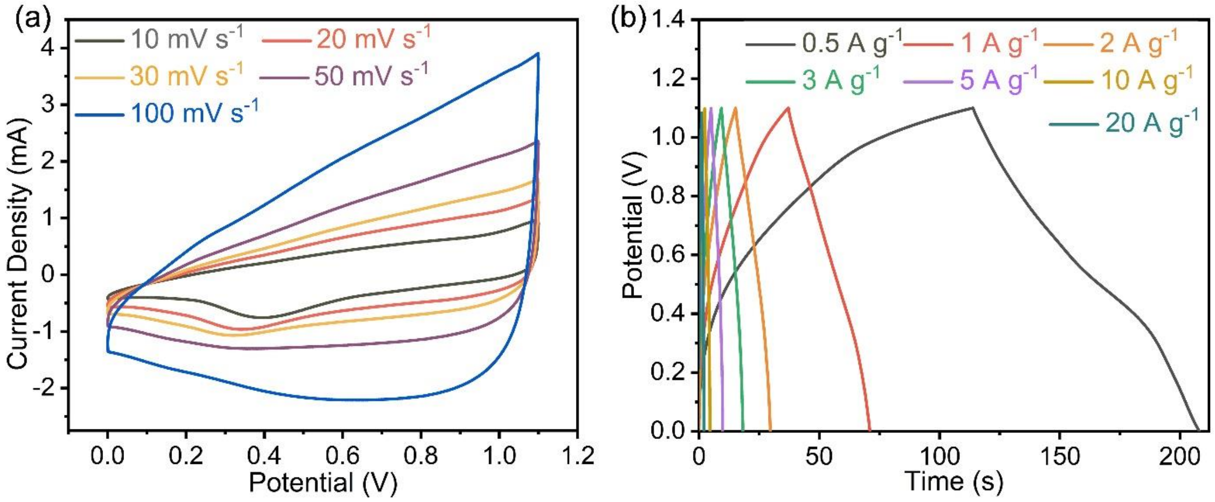

Figure S40. a) CV curves; b) The GCD curves of Co_3O_4 QDs.

43. CV and GCD curves of the CDs

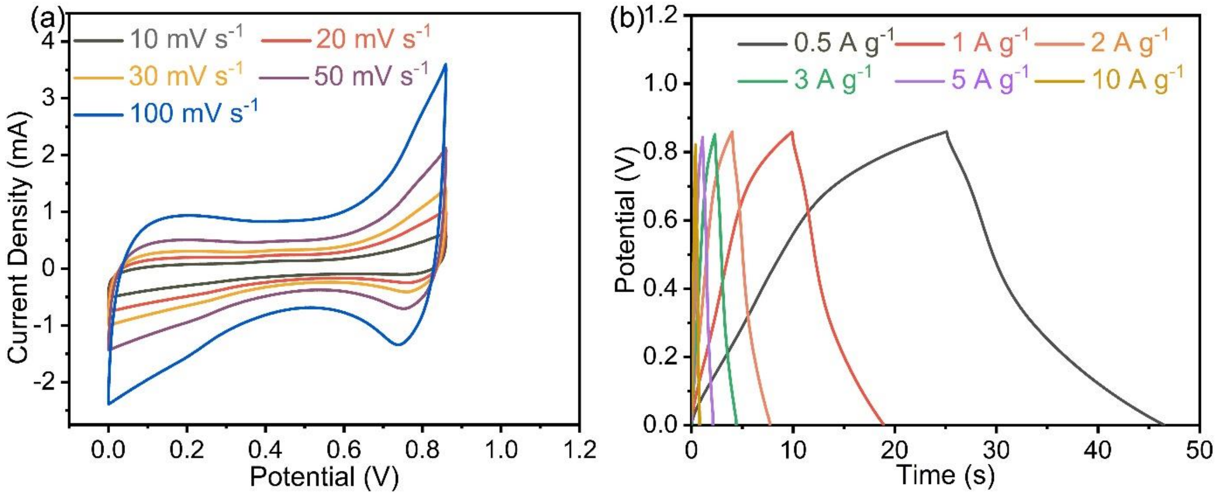

Figure S41. a) CV curves; b) The GCD curves of CDs.

44. BET and pore size distribution

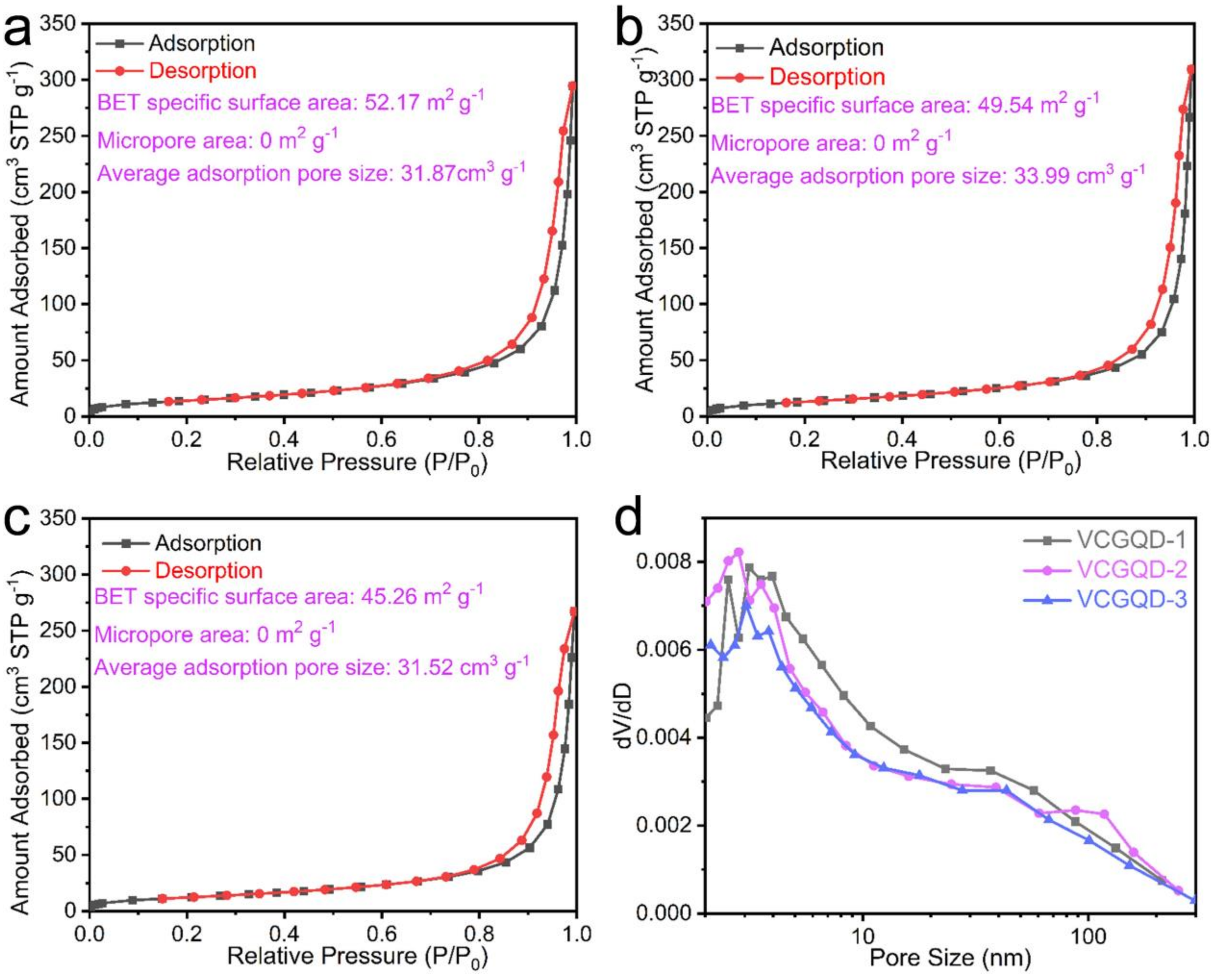

Figure S42. a) N_2 adsorption-desorption isotherms of VCGQD-1 mixed gel; b) N_2 adsorption-desorption isotherms of VCGQD-2 mixed gel; c) N_2 adsorption-desorption isotherms of VCGQD-3 mixed gel; d) the BJH adsorption branch of all mixed gel.

45. SEM image of VCGCDs electrode cross-section

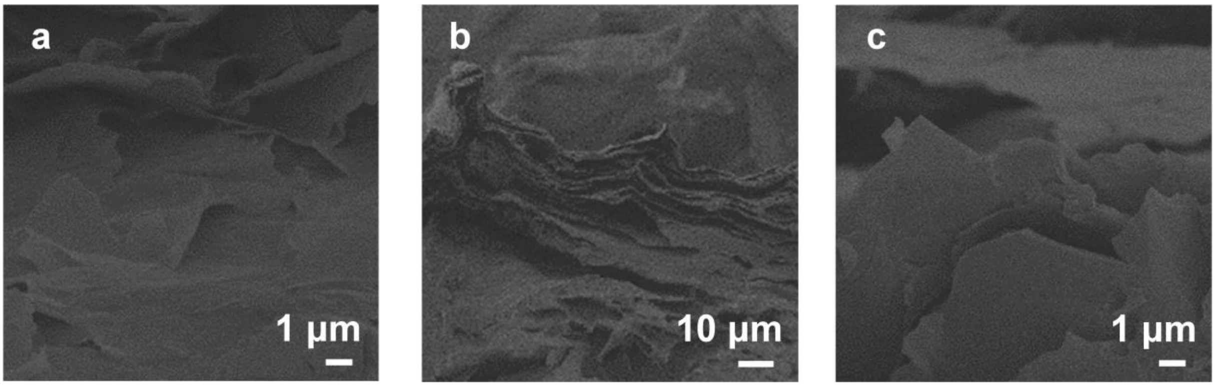

Figure S43. a, b and c) SEM images of MXene electrode cross-section at different magnification.

46. In situ XRD spectra during charging and discharging

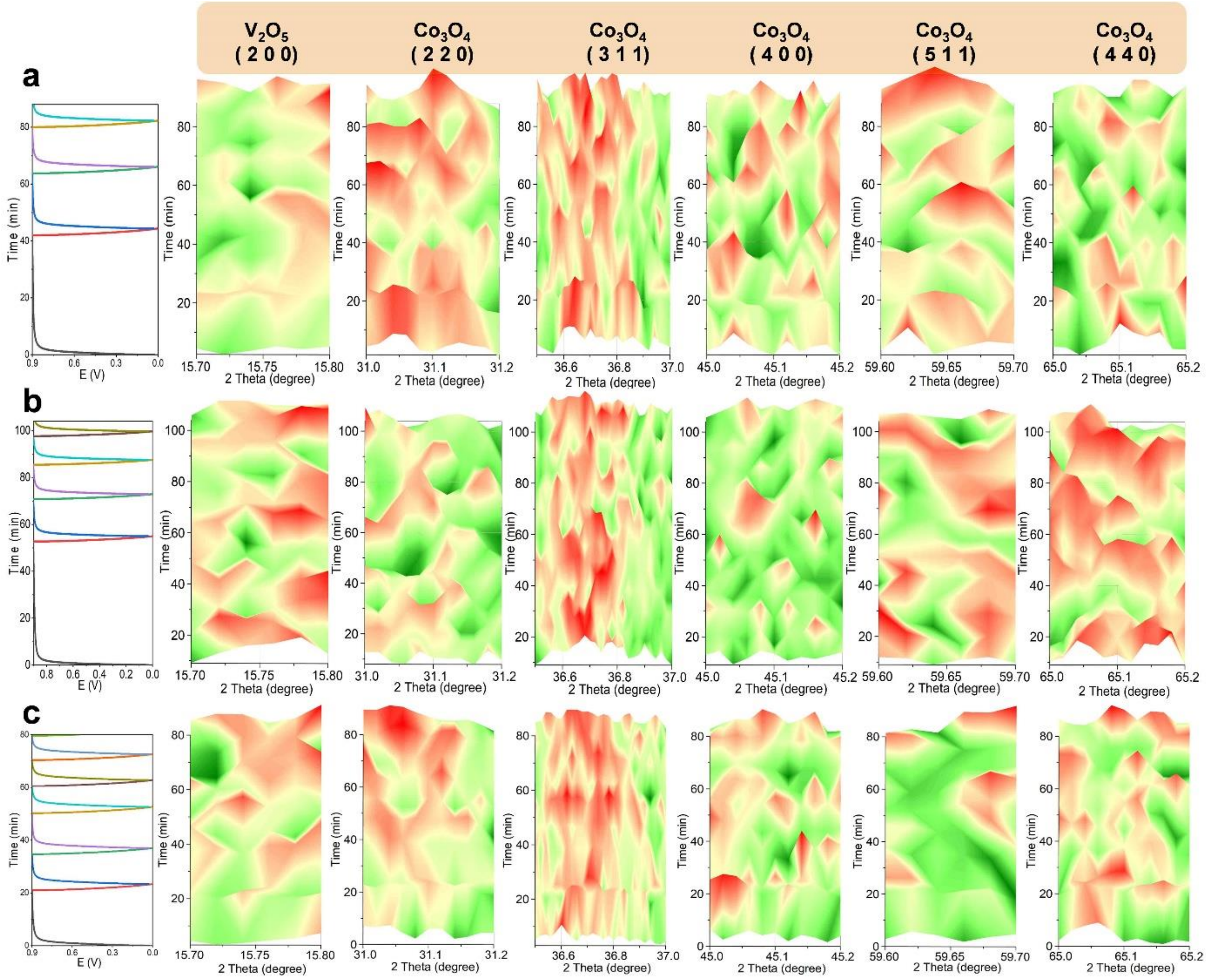

Figure S44. In situ XRD spectra during charging and discharging of a) the VCGQD-1//MXene MSCs, b) the VCGQD-2//MXene MSCs and c) the VCGQD-2//MXene MSCs.

Table S1. Comparison of electrochemical responses of relevant MSCs.

Material	Feature	Electrolyte	Scan Rate/ Current Density	Areal Capacitance (mF cm^{-2})	Power density (mW cm^{-2})	Energy density (µWh cm^{-2})	Ref.
$Ti_3C_2T_x$/ MnO_2	Hybrid film	PVA/LiCl	0.2 mA cm^{-2}	205	-	-	[1]
$Ti_3C_2T_x$/Ppy	Freestanding film	PVA/H_2SO_4	0.3 mA cm^{-2}	35.6	-	-	[2]
$Ti_3C_2T_x$/rGO	Composite aerogel	PVA/H_2SO_4	1 mV s^{-1}	34.6	0.06	2.18	[3]
$Ti_3C_2T_x$/Graphene	Spray coated film	PVA/H_3PO_4	5 mV s^{-1}	3.26	-	-	[4]
$Ti_3C_2T_x$/CNT	Freestanding film	1M KOH	2 mV s^{-1}	220	-	-	[5]
$Ti_3C_2T_x$/CNT	Printed	PVA/H_3PO_4	25 µA cm^{-2}	30.76	17.31	8.37	[6]
$Ti_3C_2T_x$/CNT	Spray coated layer by layer	1M H_2SO_4	10 mV s^{-1}	80	-	-	[7]
Mn/Mo@ MWCNT	NT	ADN/SN/LiTFSI/ PMMA	0.3 mA cm^{-2}	7.5	4.2 µWh cm^{-2}	0.3 mW cm^{-2}	[8]
$Ti_3C_2T_x$ MXene	NS	(PVA)/H_2SO_4	2 mV s^{-1}	168.1	8.4 µW h cm^{-2}	3.7 mW cm^{-2}	[9]
Ti_3C_2 MXene	NS	2 M aqueous $ZnSO_4$	0.38 mA cm^{-2}	1006.4	0.10 mWh cm^{-2}	5.90 mW cm^{-2}	[10]
MWCNT/ PANI	NT	PMMA-PC-$LiClO_4$	0.2 mA cm^{-2}	44.13	0.004 mW h cm^{-2}	0.07 mW cm^{-2}	[11]
VO_x/rGO	NS	LiCl–PVA	0.63 mA cm^{-2}	207.9	73.9 µWh cm^{-2}	3.77 mW cm^{-2}	[12]
MXene-AgNWMnONW-C60	nanocomposite	PVA-KOH	10 mV s^{-1}	216.2	19.2 µWh cm^{-2}	58.3 mW cm^{-2}	[13]
MWNTs	NT	PEGDA/[EMIM][TFSI]	0.006 mA cm^{-2}	0.51	0.34 µWh cm^{-2}	0.006 mW cm^{-2}	[14]
3DBCN	microspheres	PVA/KOH	0.05 mA cm^{-2}	41.6	0.00832 mW h cm^{-2}	0.183 mW cm^{-2}	[15]
MoS_2/PEDOT:PSS	interconnected network	PVA–H_3PO_4	0.5 mA cm^{-2}	203	50 µWh cm^{-2}	0.5 mW cm^{-2}	[16]
laser-induced graphene red-mud	Nanofbers and NP	PVDF–HFP	0.1 mA cm^{-2}	203	51 µWh cm^{-2}	0.14 mW cm^{-2}	[17]
GCP-MSCs	wrinkled micro-structured and robust interconnected	(PVA)/H_3PO_4	5 mV s^{-1}	107.5	~ 0.54 µW h cm^{-2}	~ 1.22 mW cm^{-2}	[18]
CNT/Ppy	NT	LiCl/PVA/MPII	100 µA cm^{-2}	5.17	0.44 µWh cm^{-2}	176.5 µW cm^{-2}	[19]
graphene/ MnOx/propylene	3D network	PVA/H_2SO_4	10 mV s^{-1}	13.8	1 µWh cm^{-2}	10 mW cm^{-2}	[20]

PANI/rGO/Au/PDMS	3D network	PVA/H_2SO_4	0.2 mA cm^{-2}	4.06	1.07 mW cm^{-3}	9.66 W cm^{-3}	[21]
V_2O_5 NWs/MWCNTs	NW	(PVA)/KOH	1 mA cm^{-2}	152.7	54.3 μWh cm^{-2}	801.4 μW cm^{-2}	[22]
$Ti_3C_2T_x$ MXene	NS	PVA/H_2SO_4	25 μA cm^{-2}	61	0.76 μWh cm^{-2}	0.33 mW cm^{-2}	[23]
$Ti_3C_2T_x$	NS	PVA/H_2SO_4	1.7 mA cm^{-2}	2100	0.0244 mWh cm^{-2}	0.64 mW cm^{-2}	[24]

Reference

[1] J. Zhou, J. Yu, L. Shi, Z. Wang, H. Liu, B. Yang, C. Li, C. Zhu, J. Xu, Small 2018, 14, 1.

[2] M. Zhu, Y. Huang, Q. Deng, J. Zhou, Z. Pei, Q. Xue, Y. Huang, Z. Wang, H. Li, Q. Huang, C. Zhi, Adv. Energy Mater. 2016, 6.

[3] Y. Yue, N. Liu, Y. Ma, S. Wang, W. Liu, C. Luo, H. Zhang, F. Cheng, J. Rao, X. Hu, J. Su, Y. Gao, ACS Nano 2018, 12, 4224.

[4] H. Li, Y. Hou, F. Wang, M. R. Lohe, X. Zhuang, L. Niu, X. Feng, Adv. Energy Mater. 2017, 7, 2.

[5] Q. Fu, X. Wang, N. Zhang, J. Wen, L. Li, H. Gao, X. Zhang, J. Colloid Interface Sci. 2018, 511, 128.

[6] J. Zhao, Y. Zhang, Y. Huang, X. Zhao, Y. Shi, J. Qu, C. Yang, J. Xie, J. Wang, L. Li, Q. Yan, S. Hou, C. Lu, X. Xu, Y. Yao, J. Mater. Chem. A 2019, 7, 972.

[7] Z. Zhou, W. Panatdasirisuk, T. S. Mathis, B. Anasori, C. Lu, X. Zhang, Z. Liao, Y. Gogotsi, S. Yang, Nanoscale 2018, 10, 6005.

[8] G. Lee, J. W. Kim, H. Park, J. Y. Lee, H. Lee, C. Song, S. W. Jin, K. Keum, C.-H. Lee, J. S. Ha, ACS Nano 2019, 13, 855.

[9] J. Orangi, F. Hamade, V. A. Davis, M. Beidaghi, ACS Nano 2020, 14, 640.

[10] Z. Fan, J. Jin, C. Li, J. Cai, C. Wei, Y. Shao, G. Zou, J. Sun, ACS Nano 2021, 15, 3098.

[11] L. Li, Z. Lou, W. Han, D. Chen, K. Jiang, G. Shen, Adv. Mater. Technol. 2017, 2, 1600282.

[12] K. Shen, J. Ding, S. Yang, Adv. Energy Mater. 2018, 8, 1800408.

[13] X. Li, H. Li, X. Fan, X. Shi, J. Liang, Adv. Energy Mater. 2020, 10, 1903794.

[14] H. Kim, J. Yoon, G. Lee, S. Paik, G. Choi, D. Kim, B.-M. Kim, G. Zi, J. S. Ha, ACS Appl. Mater. Interfaces 2016, 8, 16016.

[15] D. Tu, Z. Wu, J. Xu, Y. Zhou, W. Yang, Y. Yang, X. Zha, L. Shi, ACS Appl. Mater. Interfaces 2020, 12, 47416.

[16] Y. Chao, Y. Ge, Z. Chen, X. Cui, C. Zhao, C. Wang, G. G. Wallace, ACS Appl. Mater. Interfaces 2021, 13, 7285.

[17] G. Bhattacharya, S. J. Fishlock, A. Pritam, S. Sinha Roy, J. A. McLaughlin, Adv. Sustain. Syst. 2020, 4, 1900133.

[18] H. Xiao, Z.-S. Wu, F. Zhou, S. Zheng, D. Sui, Y. Chen, X. Bao, Energy Storage Mater. 2018, 13, 233.

[19] J. Yun, C. Song, H. Lee, H. Park, Y. R. Jeong, J. W. Kim, S. W. Jin, S. Y. Oh, L. Sun, G. Zi, J. S. Ha, Nano Energy 2018, 49, 644.

[20] S. Sollami Delekta, M.-M. Laurila, M. Mäntysalo, J. Li, Nano-Micro Lett. 2020, 12, 40.

[21] S. Park, H. Lee, Y.-J. Kim, P. S. Lee, NPG Asia Mater. 2018, 10, 959.

[22] J. Zhao, H. Lu, Y. Zhang, S. Yu, O. I. Malyi, X. Zhao, L. Wang, H. Wang, J. Peng, X. Li, Y. Zhang, S. Chen, H. Pan, G. Xing, C. Lu, Y. Tang, X. Chen, Sci. Adv. 2021, 7, eabd6978 15.

[23] C. J. Zhang, M. P. Kremer, A. Seral-Ascaso, S.-H. Park, N. McEvoy, B. Anasori, Y. Gogotsi, V. Nicolosi, Adv. Funct. Mater. 2018, 28, 1705506.

[24] W. Yang, J. Yang, J. J. Byun, F. P. Moissinac, J. Xu, S. J. Haigh, M. Domingos, M. A. Bissett, R. A. W. Dryfe, S. Barg, Adv. Mater. 2019, 31, 1902725.
